# Pyk2-dependent phosphorylation of LSR enhances localization of LSR and tricellulin at tricellular tight junctions

**DOI:** 10.1371/journal.pone.0223300

**Published:** 2019-10-01

**Authors:** Daiki Nakatsu, Fumi Kano, Naeko Shinozaki-Narikawa, Masayuki Murata

**Affiliations:** 1 Cell Biology Center, Institute of Innovative Research, Tokyo Institute of Technology, Nagatsuta, Midori-ku, Yokohama, Kanagawa, Japan; 2 Department of Life Sciences, Graduate School of Arts and Sciences, The University of Tokyo, Komaba, Meguro-ku, Tokyo, Japan; Thomas Jefferson University, UNITED STATES

## Abstract

Tight junctions (TJs) are cellular junctions within the mammalian epithelial cell sheet that function as a physical barrier to molecular transport within the intercellular space. Dysregulation of TJs leads to various diseases. Tricellular TJs (tTJs), specialized structural variants of TJs, are formed by multiple transmembrane proteins (e.g., lipolysis-stimulated lipoprotein receptor [LSR] and tricellulin) within tricellular contacts in the mammalian epithelial cell sheet. However, the mechanism for recruiting LSR and tricellulin to tTJs is largely unknown. Previous studies have identified that tyrphostin 9, the dual inhibitor of Pyk2 (a nonreceptor tyrosine kinase) and receptor tyrosine kinase platelet-derived growth factor receptor (PDGFR), suppresses LSR and tricellulin recruitment to tTJs in EpH4 (a mouse mammary epithelial cell line) cells. In this study, we investigated the effect of Pyk2 inhibition on LSR and tricellulin localization to tTJs. Pyk2 inactivation by its specific inhibitor or repression by RNAi inhibited the localization of LSR and downstream tricellulin to tTJs without changing their expression level in EpH4 cells. Pyk2-dependent changes in subcellular LSR and tricellulin localization were independent of c-Jun N-terminal kinase (JNK) activation and expression. Additionally, Pyk2-dependent LSR phosphorylation at Tyr-237 was required for LSR and tricellulin localization to tTJs and decreased epithelial barrier function. Our findings indicated a novel mechanism by which Pyk2 regulates tTJ assembly and epithelial barrier function in the mammalian epithelial cell sheet.

## Introduction

The mammalian epithelial cell sheet contains at least six types of cellular junctions: tight junctions (TJs), adherens junctions, desmosomes, hemidesmosomes, focal adhesions, and gap junctions [1–3]. Dysregulation of any of these cellular junctions causes mammalian epithelial cell sheet dysfunction, which, in turn, causes various diseases [2]. In the mammalian epithelial cell sheet, TJs regulate molecular transport within the intercellular space and separate compartments of proteins and lipids localized to apical and basolateral membranes [4,5]. Dysregulation of TJs also causes various diseases of the vascular system, gastrointestinal tract, liver, and respiratory tract and other viral infections [6,7]. Tricellular TJs (tTJs) are generated within tricellular contacts (TCs) in the mammalian epithelial cell sheet and comprise multiple transmembrane proteins (e.g., lipolysis-stimulated lipoprotein receptor [LSR], immunoglobulin-like domain-containing receptor 1 [ILDR1], ILDR2, and tricellulin) [8–10]. LSR is a single-pass transmembrane protein mainly expressed in the epididymis, gall bladder, liver, lungs, nasal mucosa, small intestine, and skin [10], while ILDR1, ILDR2, and tricellulin are also expressed in specific tissues [8,10,11]. Tissue-specific combinations of tTJ proteins are believed to generate different barrier properties of tTJs and affect molecular transport through TCs. This belief is supported by the finding that switching from LSR to ILDR1 or ILDR2 in tTJs decreases paracellular barrier function of the mouse mammary epithelial cell line EpH4 [10]. In addition to the expression pattern of tTJ proteins, their recruitment to tTJs is also considered important for barrier function. Studies have reported several mechanisms for tTJ protein recruitment to tTJs. For example, LSR, ILDR1, or ILDR2 localization to tTJs or expression of claudin‐1 or occludin (components of bicellular TJs [bTJs]) increases tricellulin recruitment to tTJs [9,10,12,13]. Several studies have also reported that serine/threonine kinase controls tTJ protein recruitment to tTJs. For example, c-Jun N-terminal kinase (JNK) activation by anisomycin recruits tricellulin from tTJs to surrounding nuclei in the cytoplasmic region [14]. A decrease in JNK1 or JNK2 expression inhibits LSR and tricellulin recruitment to tTJs through dephosphorylation of serine 288 on LSR [15]. A recent study has reported that cytosolic tyrosine kinase enhances tricellular junction component protein recruitment to TCs in *Drosophila* cells [16].

In this study, we focused on the Pyk2, which is a nonreceptor tyrosine kinase, because we previously identified tyrphostin 9, the dual inhibitor of Pyk2 and receptor tyrosine kinase platelet-derived growth factor receptor (PDGFR), suppresses LSR and tricellulin recruitment to tTJs in EpH4 cells [15]. We found that an inhibitor against Pyk2 and Pyk2‐knockdown by RNA interference (RNAi) in EpH4 cells disperses LSR and tricellulin from tTJs to bTJs and cytoplasmic regions. Furthermore, we identified that Pyk2-dependent LSR phosphorylation at Tyr-237 is required to localize LSR and tricellulin to tTJs and decrease epithelial barrier function.

## Materials and methods

### Cell culture

EpH4 cells were kindly gifted by Dr. Ernst Reichmann (University Children’s Hospital Zurich, Zurich, Switzerland), and EpH4-Cl3 cells (EpH4 cells that stably express the bTJ protein claudin‐3 fused to the yellow fluorescence protein [Cl3-YFP]) were kindly gifted by Dr. Mikio Furuse (A National Institute for Physiological Sciences, Aichi, Japan) [17]. EpH4 cells were cultured in Dulbecco’s Modified Eagle's Medium (DMEM; Nissui, Tokyo, Japan) that contained 10% fetal calf serum (FCS; GIBCO, Ireland), penicillin (GIBCO), and streptomycin (GIBCO). EpH4-Cl3 cells were cultured in DMEM that contained 10% FCS, penicillin, streptomycin, and geneticin (Invitrogen, CA, USA).

### Antibodies and reagents

The following primary and secondary antibodies were used in this study: rat anti‐LSR antibody (A kind gift from Dr. Mikio Furuse, A National Institute for Physiological Sciences, Aichi, Japan, [10]), mouse anti‐ZO‐1 antibody (A kind gift from Dr. Mikio Furuse, A National Institute for Physiological Sciences, Aichi, Japan, [18]), mouse anti‐JNK1 antibody (Cell Signaling Technology, Inc., MA, USA), rabbit anti‐Pyk2 antibody (Abcam, Cambridge, UK), rabbit anti‐phospho-Pyk2 (Tyr-402) antibody (Cell Signaling Technology), rabbit anti‐tricellulin antibody (A kind gift from Dr. Mikio Furuse, A National Institute for Physiological Sciences, Aichi, Japan, [8]), rabbit anti‐SAPK/JNK antibody (Cell Signaling Technology), rabbit anti‐phospho‐SAPK/JNK (Thr-183/Tyr-185) antibody (Cell Signaling Technology), rabbit anti‐JNK2 antibody (Cell Signaling Technology), rabbit anti‐JNK3 antibody (Cell Signaling Technology), rabbit anti-phospho-tyrosine antibody (Abcam), rabbit anti‐green fluorescent protein (GFP) antibody (Medical and Biological Laboratories Co., Japan), rabbit anti‐phospho-focal adhesion kinase (FAK) (Tyr-397) antibody (Cell Signaling Technology), normal rabbit IgG antibody (Cell Signaling Technology), horseradish peroxidase (HRP)‐conjugated anti‐rat antibody (Chemicon International Inc., CA, USA), HRP‐conjugated anti‐rabbit antibody (Chemicon International Inc.), HRP‐conjugated anti‐mouse antibody (Upstate Biotechnology, NY, USA), Cy3‐conjugated anti‐rat antibody (Kirkegaard and Perry Laboratories Inc, MD, USA), Cy3‐conjugated anti‐rabbit antibody (Chemicon International Inc.), Alexa Fluor 546-conjugated anti-rabbit antibody (Molecular Probes, OR, USA) and Alexa Fluor 647-conjugated anti-mouse antibody (Molecular Probes). PF-431396 (PF-43) was purchased from Sigma-Aldrich Corporation (MO, USA) and GSK2256098 was purchased from Selleck Chemicals (TX, USA). These inhibitors diluted to appropriate concentrations in dimethyl sulfoxide (DMSO), and stored at −20°C.

### siRNA transfection

Short interfering RNA (siRNA) transfection assays using EpH4‐Cl3 cells were performed, as described previously [15] but with slight modifications. Briefly, EpH4‐Cl3 cells were transfected with 100 pmol of mouse Pyk2‐targeting siRNA (Mm_Ptk2b_1911; Sigma) or scrambled siRNA (Ambion, Inc., TX, USA) using Lipofectamine^®^ RNAiMax Reagent (Invitrogen) according to the manufacturer’s instructions. After incubation for 24–72 h, the cells were used for immunofluorescence, immunoblotting, and transepithelial electrical resistance (TER) measurement assays.

### Plasmid vector transfection

A plasmid encoding LSR‐GFP was established by introducing the mouse *LSR* gene into the pEGFP-N1 vector (Clontech Laboratories, CA, USA), as described previously [15]. A plasmid encoding LSR(Y237F)‐GFP was generated by polymerase chain reaction (PCR)‐based site‐directed mutagenesis using the primers, 5’-TGCCCTGAGGCCCTTTTCGCTGCTGGCAAAGCAGCCACC-3’ and 5’-TGCTTTGCCAGCAGCGAAAAGGGCCTCAGGGCAACAGC-3’. Transfection of the plasmid into EpH4 cells was performed as described previously [15] but with slight modifications. Briefly, EpH4 cells were transfected with 1 μg/ml of the plasmid encoding LSR‐GFP or LSR(Y237F)‐GFP using Lipofectamine^®^ LTX (Invitrogen) according to the manufacturer’s instructions. After incubation for 24–72 h, the cells were used for immunofluorescence, immunoblotting, transfection efficiency measurement and TER measurement assays.

### Cotransfection of siRNA and plasmid vector

Cotransfection assays of siRNA and the plasmid vector using EpH4 cells were carried out as described previously [15] but with slight modifications. Briefly, mouse Pyk2‐targeting siRNA, scrambled siRNA, and the plasmid encoding LSR‐GFP or LSR(Y237F)‐GFP were used for cotransfection assays. EpH4 cells were cotransfected with 12 μg of the expression plasmid vector and 100 pmol of siRNA using Lipofectamine^®^ 2000 (Invitrogen) according to the manufacturer’s instructions. After incubation for 72 h, the cells were used for immunoprecipitation and immunoblotting assays.

### Immunofluorescence

For staining of LSR, tricellulin, and ZO‐1, EpH4 cells were fixed with 1% paraformaldehyde (PFA) in phosphate-buffered saline (PBS) for 15 min at room temperature and then washed with PBS. Subsequently, the cells were incubated with 0.2% Triton X‐100 in PBS for 15 min. After incubation, the cells were washed with PBS and then blocked with PBS containing 5% nonfat dry milk. The blocked cells were incubated with primary antibodies for 12–24 h at 4°C and then visualized with fluorescence‐conjugated secondary antibodies for 1 h at room temperature. The cells were observed under an LSM 710 confocal microscope equipped with a 63x Plan‐NEOFLUAR oil immersion objective (Carl Zeiss, Jena, Germany) or A1 confocal microscope equipped with a 60x Plan-Apochromat water immersion objective (Nikon, Tokyo, Japan).

### Identification of three-dimensional subcellular localization of LSR or tricellulin

First, EpH4 or EpH4-Cl3 cells were immunostained with anti-LSR, anti-tricellulin or anti-ZO-1 antibodies and counterstained with DAPI. Z-stack images of the cells (1024 × 1024 pixels, 18 layers, 1 μm step with overlay) were taken using Nikon A1 confocal microscopy. Three-dimensional images of the cells were reconstructed from the z-stack images using Nikon NIS-Elements software. Three-dimensional subcellular localization of tTJ proteins in the cells were identified using the images.

### Quantification of dispersion of LSR or tricellulin from the tTJs to the bTJs

We quantified the dispersion of LSR or tricellulin from the tTJs to the bTJs in EpH4 and EpH4-Cl3 cells as described previously [15], with a slight modification. We acquired the fluorescent images of cells using A1 confocal microscopy (Nikon) and measured the fluorescence intensity of LSR and tricellulin at bTJs and tTJs using NIS-Elements software (Nikon).

### Immunoprecipitation

Immunoprecipitation assays using EpH4 cells were carried out as described previously [19] but with slight modifications. First, confluent LSR‐GFP- or LSR(Y237F)‐GFP-transfected EpH4 cells were washed with ice‐cold PBS and incubated with ice‐cold radioimmunoprecipitation assay (RIPA) buffer (TBS containing 1% NP‐40, 0.1% sodium dodecyl sulfate [SDS], and 0.5% sodium deoxycholate) containing a protease inhibitor mixture (Roche, Basel, Switzerland) and PhosSTOP phosphatase inhibitor mixture (Roche) for 10 min at 4°C. After incubation, the cells were scraped from culture dishes and passed 15 times through a 27‐gage needle. The cell lysates were incubated for 30 min at 4°C and, then, centrifuged at 20,400× *g* for 1 min. The supernatants were treated with rabbit anti‐GFP antibody for 12 h at 4°C, and then the supernatant and antibody mixtures were incubated with Protein G Sepharose 4 Fast Flow beads (GE Healthcare) for 60 min at 4°C. After incubation, the supernatant, antibody, and bead mixtures were centrifuged at 20,400× *g* for 5 s. The precipitates were washed with RIPA buffer thrice, and the pellet was resuspended in 2 × SDS sample buffer, boiled for 5 min at 100°C, and analyzed with sodium dodecyl sulfate–polyacrylamide gel electrophoresis (SDS-PAGE) and immunoblotting.

### SDS‐PAGE and immunoblotting

SDS-PAGE was performed using SDS-polyacrylamide gradient gels (5%–20% acrylamide) and gel running buffer (0.25 M Tris-base, 1.92 M glycine, and 0.1% SDS) under constant current conditions (20 mA/gel) for 80 min at room temperature. Then, a Trans-Blot SD cell (Bio-Rad Laboratories, Inc., CA, USA) was used to transfer proteins to a polyvinylidene fluoride (PVDF) membrane. After the transfer, the PVDF membrane was blocked with 5% bovine serum albumin (BSA) or 5% nonfat dry milk in TBST (TBS containing 0.1% Tween-20) for 60 min at room temperature and incubated with a primary antibody dissolved in TBST containing 5% BSA or 5% nonfat dry milk for 12–24 h at 4°C. Subsequently, the PVDF membrane was incubated with a secondary antibody dissolved in TBST containing 5% BSA or 5% nonfat dry milk for 60 min at room temperature. Then, a Western Lightning Plus-ECL chemiluminescence detection kit (PerkinElmer, Inc., MA, USA) was used to detect signals of antigen–antibody complexes, and a LAS-4000 mini imaging system (GE Healthcare, IL, USA) was used to quantify the signal intensities.

### TER measurement

EpH4-Cl3 cells were plated onto Transwell filters and after the cells were treated with PF-43 or DMSO. EpH4-Cl3 cells also were transfected with scrambled siRNA (control) or siRNAs targeted to messenger RNAs (mRNAs) encoding Pyk2 (Pyk2 KD) and plated onto Transwell filters. EpH4 cells were transfected with plasmid vectors expressing LSR-GFP or LSR(Y237F)-GFP and plated onto Transwell filters. After 24, 48, and 72 h, the culture medium was changed to new DMEM supplemented with 10% FCS. Before the medium change, TER was measured using a Millicell-ERS epithelial volt-ohm meter (Millipore Corporation, MA, USA).

### Statistical analysis

Data analysis was conducted using the F-test and Student’s or Welch’s *t*-test. Results were reported as mean ± standard error of the mean (SEM). *P* < 0.05 was considered statistically significant.

## Results

### PF-43 treatment inhibited LSR and tricellulin localization to tTJs without changing their expression level

**Fig 1:**
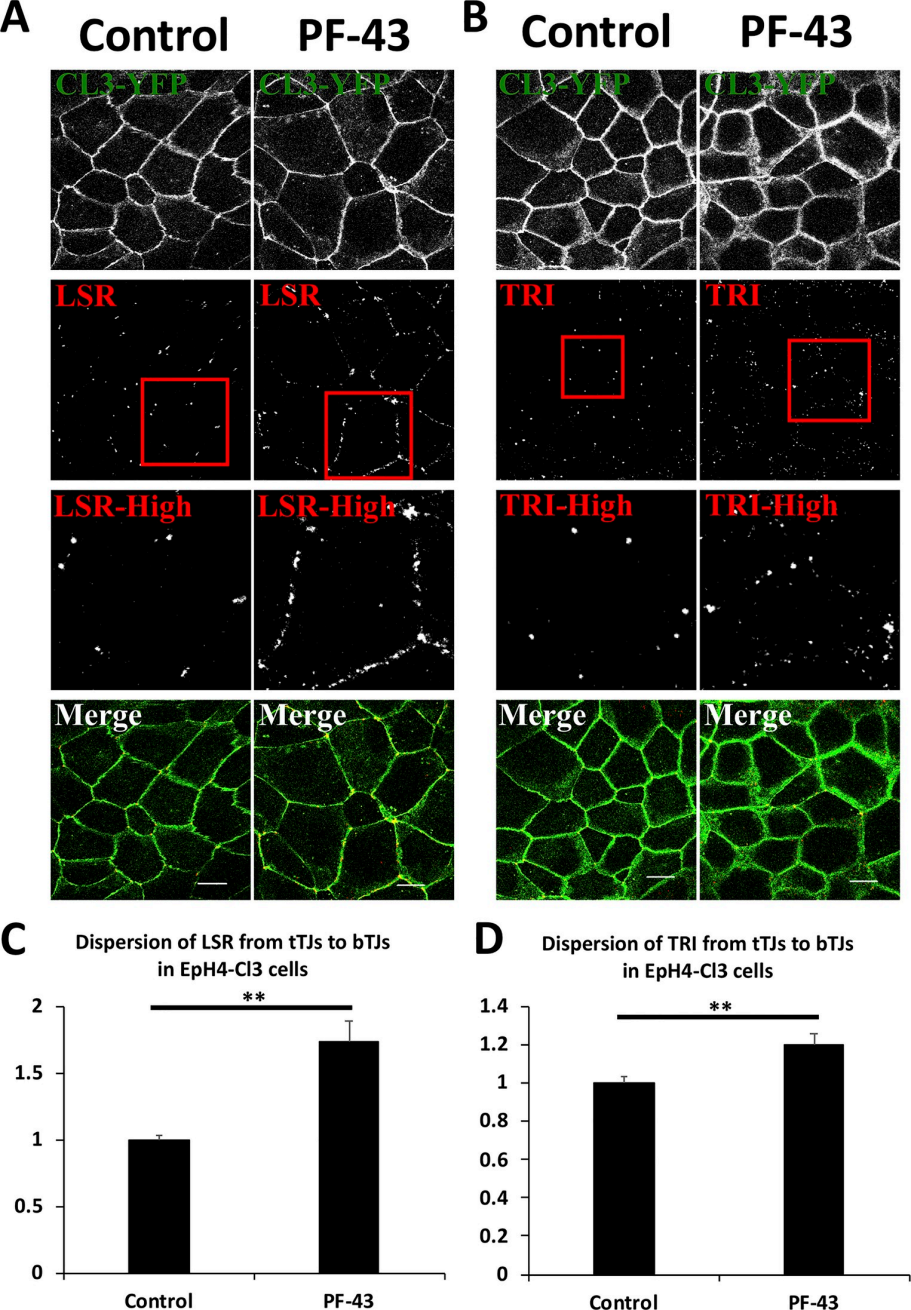
Subcellular localization of tTJ proteins in PF-43-treated EpH4-Cl3 cells. EpH4-Cl3 cells were incubated with DMSO (Control) or 20 μM PF-43 for 120 min. The cells were then immunostained with anti-LSR (A) and anti-tricellulin (B, TRI) antibodies, and observed using confocal microscopy. The red rectangular regions represent higher magnifications (LSR-High and TRI-High). Merge represents the merged image. Scale bar = 10 μm. Dispersion of LSR (C) and TRI (D) in Control or 20 μM PF-43 treated cells was calculated, and the means and the SEMs are shown in the graph (*n* = 30, **p < 0.01). The dispersion values in control cells were set to 1.

We previously identified tyrphostin 9, which is a dual inhibitor of Pyk2 and PDGFR, treatment suppresses LSR and tricellulin localization to tTJs in the mammalian epithelial cell sheet, while AG-370, AG-494, AG-1296, tyrphostin 46, and tyrphostin 51, which are inhibitors of PDGFR, have little or no effect on LSR localization to tTJs [15]. From these results, we assumed that Pyk2 would regulate LSR and tricellulin localization to tTJs. To test this hypothesis, we investigated more closely the effect of Pyk2 inhibition on localization of LSR and tricellulin at the tTJs. We used EpH4-Cl3 cells because tTJs were visualized as intersections of the three-way junction of Cl3-YFP-positive TJs. Confluent EpH4-Cl3 cells were treated with DMSO or 20 μM PF-43, which is a Pyk2 inhibitor, for 2 h. After the cells were fixed, they were subjected to immunofluorescence using anti-LSR or anti-tricellulin antibodies and observed by LSM710 confocal microscopy. We chose concentrations of PF-43 according to previous studies [20,21]. As Fig 1A and 1B show, some of the LSR and tricellulin diffused from tTJs into bTJs in PF-43-treated cells, whereas in DMSO-treated cells, the tTJ proteins remained localized to tTJs. Though we occasionally observed the LSR or tricellulin-positive dots in the cytoplasmic region (Fig 1A and 1B), the dispersion of LSR and tricellulin to bTJs was the biggest influence caused by PF-43. We measured the fluorescence intensity of LSR and tricellulin at bTJs and tTJs, and quantified their dispersion as described in Materials and Methods. The results indicated that both LSR and tricellulin were significantly localized to bTJs in PF-43 treated cells (Fig 1C and 1D).

**Fig 2:**
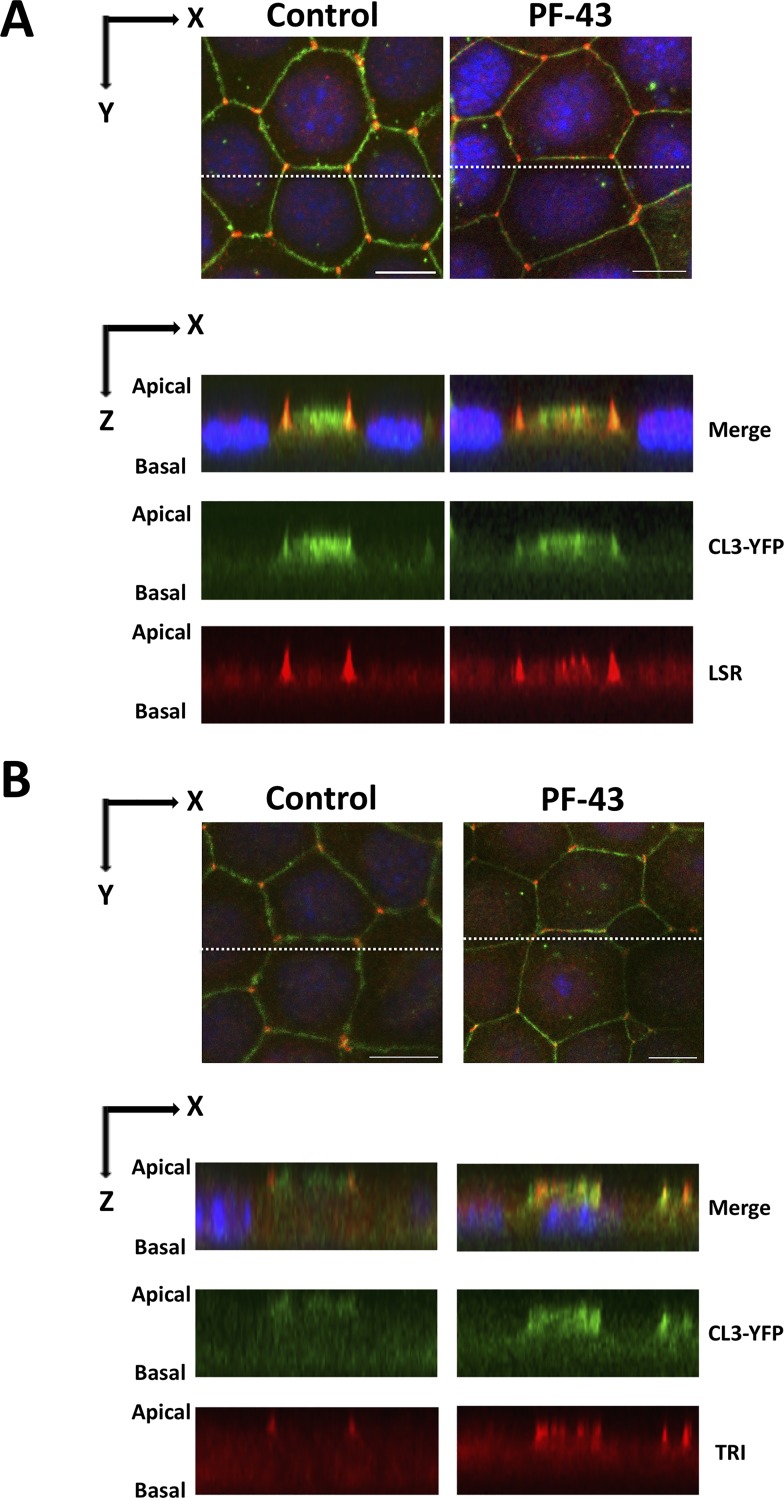
Three-dimensional subcellular localization of LSR and tricellulin in PF-43-treated EpH4-Cl3 cells. EpH4-Cl3 cells were incubated under the same conditions as in Fig 1A. The cells were immunostained with anti-LSR (A, LSR) and anti-tricellulin (B, TRI) antibodies and counterstained with DAPI, and three-dimensional images of the cells were prepared as described in the Materials and Methods. We identified the three-dimensional subcellular localization of LSR and TRI in DMSO- (Control) or PF-43-treated cells from the images. Imaging along the z-axis at the indicated position (white dotted line, upper panel) shows colocalization of LSR and TRI with CL3-YFP at bTJs in PF-43-treated cells (lower panel). Bar, 10 μm.

To carefully observe their localization, we used z-stack images of PF-43 or DMSO-treated cells. The images revealed that both LSR and tricellulin were diffused to bTJs, which were labelled with CL3-YFP, but not to entire the bicellular contact sites (Fig 2A and 2B), and that most of these proteins were still observed at tTJs. In addition, the dispersed LSR or tricellulin were found as dots (Fig 2A and 2B), indicating the possibility of the self-assembly or interaction each other in bTJs. However, unfortunately, we could not determine whether the dispersed LSR and tricellulin colocalized in bTJs because the double-staining of these proteins using our antibodies caused unexpected decrease in fluorescence signal of tricellulin (S1 Fig).

We examined how the concentration dependency of PF-43 affected the localization of LSR to tTJs. The cells were treated either with PF-43 at the concentration of 10, 20, 40, or 80 μM, or with DMSO as control for 2 h. The cells were fixed, and were subjected to immunofluorescence using anti-LSR antibody. Some of the LSR diffused from tTJs into bTJs in 10 μM PF-43-treated cells, which was prominent by treatment with 20 μM PF-43 (S2A Fig). In contrast, the tTJ proteins remained localized to tTJs in DMSO-treated cells (S2A Fig). The PF-43 treatment at higher concentration (40 and 80 μM) caused the substantial disruption of both bTJs (S2A Fig, CL3-YFP) and tTJs (S2A Fig, LSR), suggesting the perturbation of whole epithelial cell adhesion. We also found that longer incubation (4 h) of 20 μM PF-43 also disrupted the epithelial cell adhesion (S2B Fig). In the following experiments, we treated cells with 20 μM PF-43 for 2 h as the mislocalization of LSR from tTJs to bTJs was most clearly visible.

**Fig 3:**
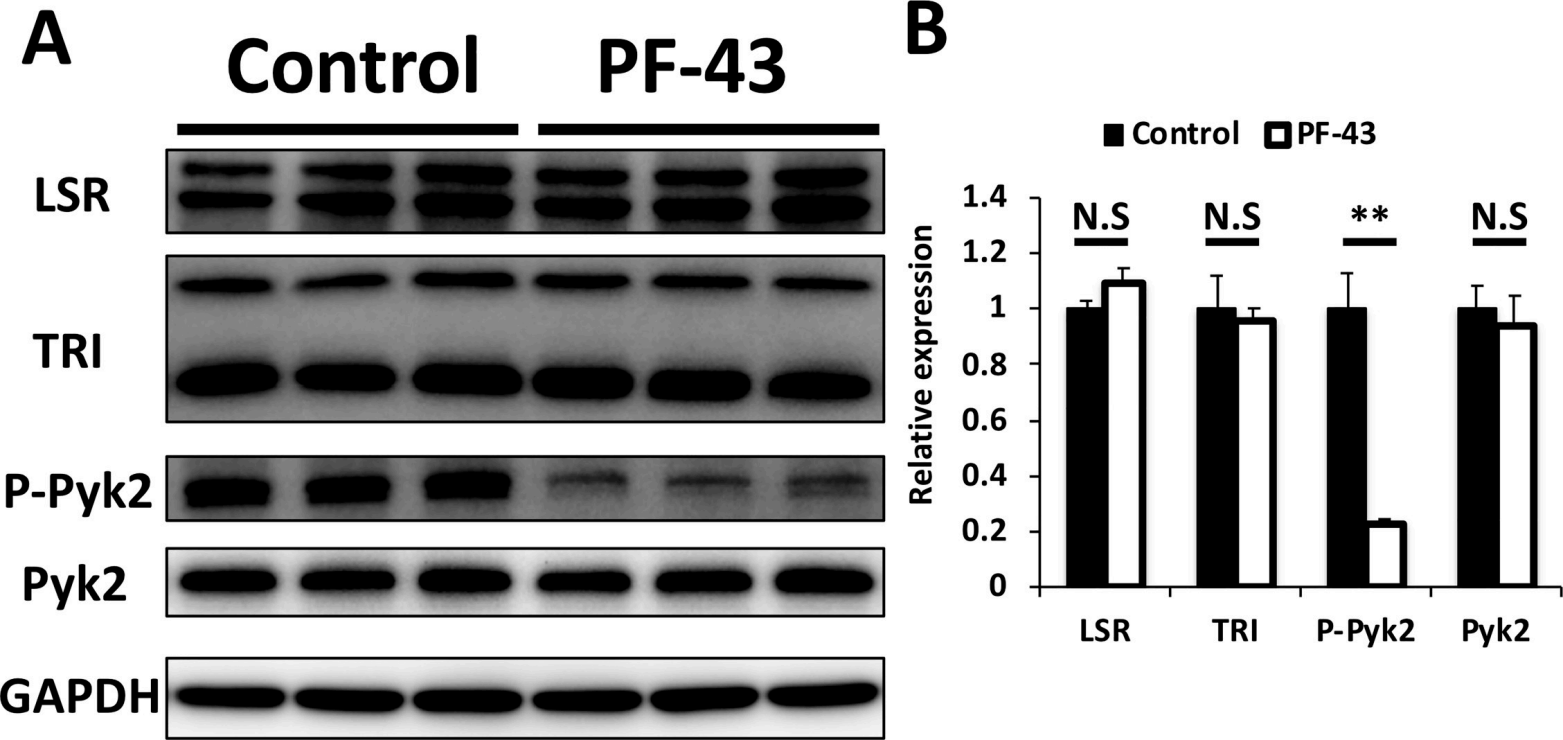
Expression of tTJ proteins in PF-43-treated EpH4-Cl3 cells. (A) EpH4-Cl3 cells were incubated with DMSO (Control) or 20 μM PF-43 for 120 min, and the extracts were subjected to immunoblotting using antibodies against LSR, tricellulin (TRI), phosphorylated Pyk2 (Tyr402) (P-Pyk2), Pyk2, and GAPDH. (B) Band intensities of LSR, TRI, P-Pyk2, and Pyk2 in (A) were measured and normalized to GAPDH expression. The expression level of each protein in control cells was set to 1. The means and SEMs are shown in the graph (*n* = 3; **p < 0.01; ^N.S.^*p* > 0.05).

Next, to confirm whether PF-43 treatment inhibits Pyk2 phosphorylation (activation), we examined its effect on the expression of tTJ proteins by immunoblotting. EpH4‐Cl3 cells were incubated with DMSO and PF-43, as described above, then lysed, and analyzed by immunoblotting using antibodies against LSR, tricellulin, phosphorylated Pyk2 at Y402, Pyk2 and glyceraldehyde 3-phosphate dehydrogenase (GAPDH), an internal control. The phosphorylated (activated) Pyk2 expression level was found to be significantly decreased in PF-43‐treated cells compared to DMSO‐treated cells (Fig 3A and 3B), while the LSR, tricellulin, and Pyk2 expression levels were not significantly changed between PF-43‐ and DMSO‐treated cells (Fig 3A and 3B).

Since PF-43 is a dual Pyk2/FAK inhibitor [22], PF-43-induced dispersion of tTJ proteins might not depend on Pyk2 but instead on FAK. To confirm this hypothesis, we used a FAK-specific inhibitor, GSK2256098 (GSK), and examined its effect on the localization of LSR and tricellulin. We found that 1 μM GSK was sufficient to inhibit the phosphorylation (activation) of FAK at Tyr397 (S3A Fig and S3C Fig), which correlates with FAK activity [23], without significant change in phosphorylation level of Pyk2 at Tyr402 (S3B Fig and S3C Fig). And LSR and tricellulin remained at tTJs in GSK-treated cells (S3D Fig and S3E Fig).

These results suggested that Pyk2 inactivation by PF-43 treatment inhibits LSR and tricellulin localization to tTJs without changing protein expression levels. Since a recent study reported little expression of ILDR1 and ILDR2 in EpH4 cells [10], dysregulation of LSR and tricellulin localization to tTJs by Pyk2 inactivation would be independent of these tTJ proteins.

### Pyk2 enhanced LSR and tricellulin localization to tTJs without changing their expression level

**Fig 4:**
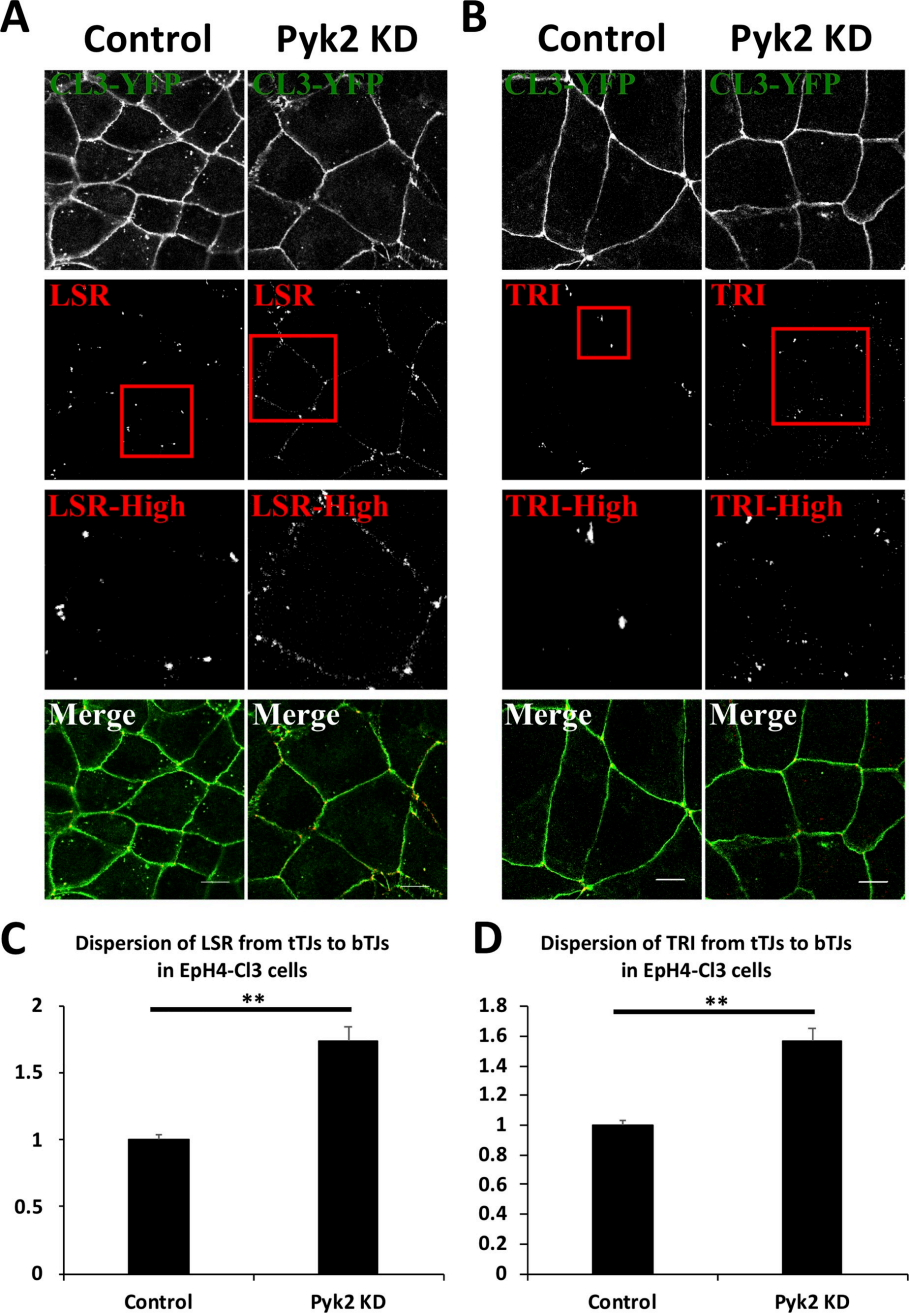
Change in localization of tTJ proteins to bTJs in Pyk2-knockdown cells. EpH4-Cl3 cells were transfected with scrambled siRNA (Control) or siRNAs targeted to mRNAs encoding Pyk2 (Pyk2 KD). After 48 h, the cells were subjected to immunofluorescence using anti-LSR (A, LSR) and anti-tricellulin (B, TRI) antibodies. The red rectangular regions represent higher magnifications (LSR-High and TRI-High). Merge represents the merged image. Scale bar = 10 μm. Dispersion of LSR (C) and TRI (D) in Control or Pyk2 KD cells was calculated, and the means and the SEMs are shown in the graph (*n* = 30, **p < 0.01). The dispersion values in control cells were set to 1.

**Fig 5:**
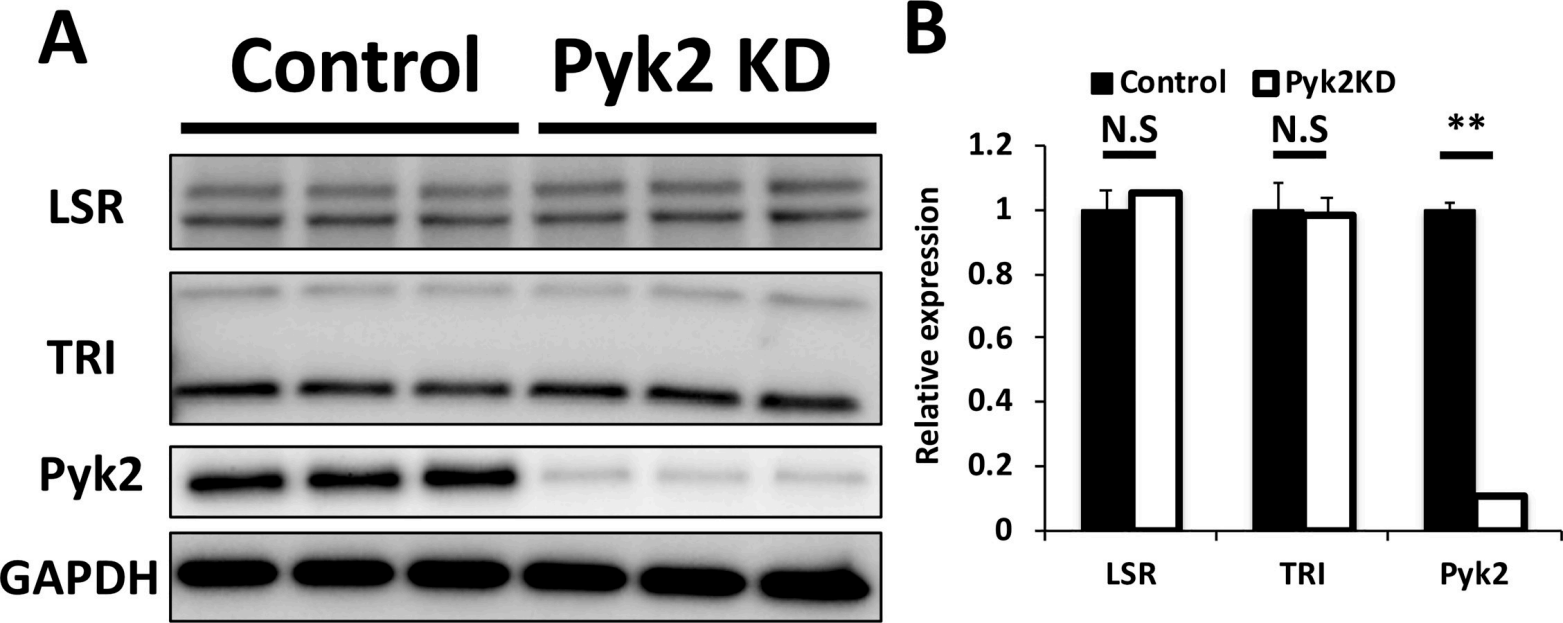
Expression of tTJ proteins in Pyk2-knockdown cells. (A) EpH4-Cl3 cells were transfected with scrambled siRNA (Control) or siRNAs targeted to mRNAs encoding Pyk2 (Pyk2 KD). After 48 h, extracts from control cells or Pyk2-knockdown EpH4-Cl3 cells were lysed and analyzed by immunoblotting using antibodies against LSR (LSR), tricellulin (TRI), Pyk2, and GAPDH. (B) Band intensities of LSR, TRI, and Pyk2 were measured and normalized to GAPDH expression. The expression levels in control cells were set to 1. The means and SEMs are shown in the graph (*n* = 3; **p < 0.01; ^N.S.^*p* > 0.05).

**Fig 6:**
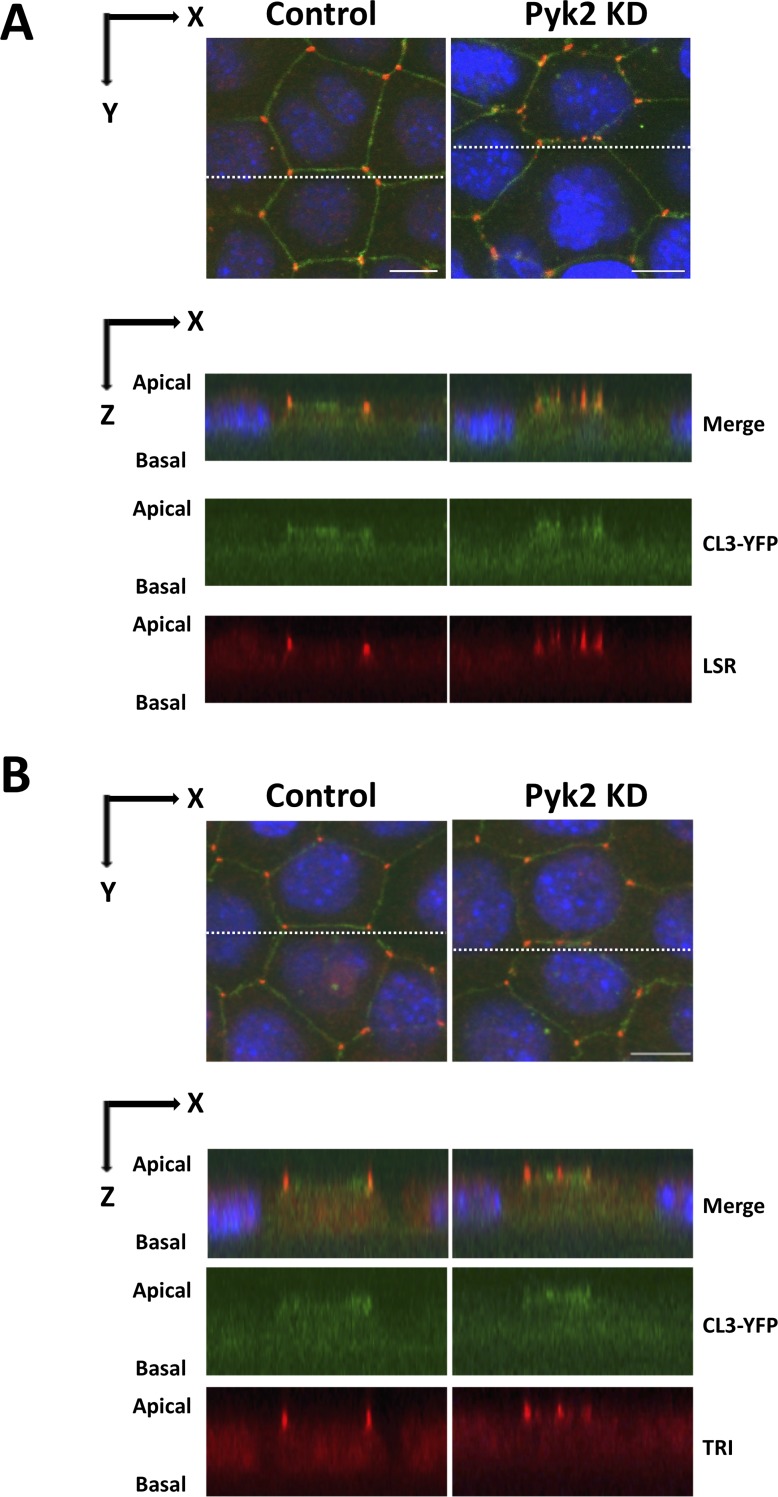
Three-dimensional subcellular localization of LSR and tricellulin in Pyk2-knockdown cells. EpH4-Cl3 cells were incubated under the same conditions as in Fig 4A. The cells were then immunostained with anti-LSR (A, LSR) and anti-tricellulin (B, TRI) antibodies and counterstained with DAPI, and three-dimensional images of the cells were prepared as described in the Materials and Methods. We identified the three-dimensional subcellular localization of LSR and TRI in scrambled siRNA—(Control) or siRNAs targeted to mRNAs encoding Pyk2- (Pyk2 KD) transfected cells using the images. Imaging along the z-axis at the indicated position (white dotted line, upper panel) reveals colocalization of LSR and TRI with CL3-YFP at bTJs in Pyk2 KD cells (lower panel). Bar, 10 μm.

We performed Pyk2 knockdown by RNAi and investigated its effects on cellular localization and expression of LSR and tricellulin. First, Pyk2-targeting siRNA or scrambled siRNA was transfected into EpH4‐Cl3 cells. After 2 days of the transfection, the cells were fixed with PFA and stained with LSR or tricellulin antibodies (Fig 4). Decrease of Pyk2 expression in response to siRNA transfection was confirmed by immunoblotting (Fig 5). Confocal microscopic analysis revealed that Pyk2 knockdown caused diffusion of LSR and tricellulin into bTJs (Fig 4A and 4B). We confirmed this result by quantifying the dispersion of LSR and tricellulin to the bTJs (Fig 4C and 4D). The dispersed LSR and tricellulin were found in the bTJs in Pyk2-knockdown cells similar to PF-43-treated cells (Fig 6A and 6B). Also, we occasionally observed a few LSR or tricellulin-positive dots in the cytoplasmic regions as well as in PF-43 treated cells (Fig 4A and 4B). Subsequently, we found that LSR and tricellulin expression was unaffected by Pyk2 knockdown (Fig 5A and 5B). These results indicated that Pyk2 activity is required for LSR and tricellulin localization to tTJs without changing their expression levels.

### Pyk2 knockdown had no significant effect on JNK activation and expression

**Fig 7:**
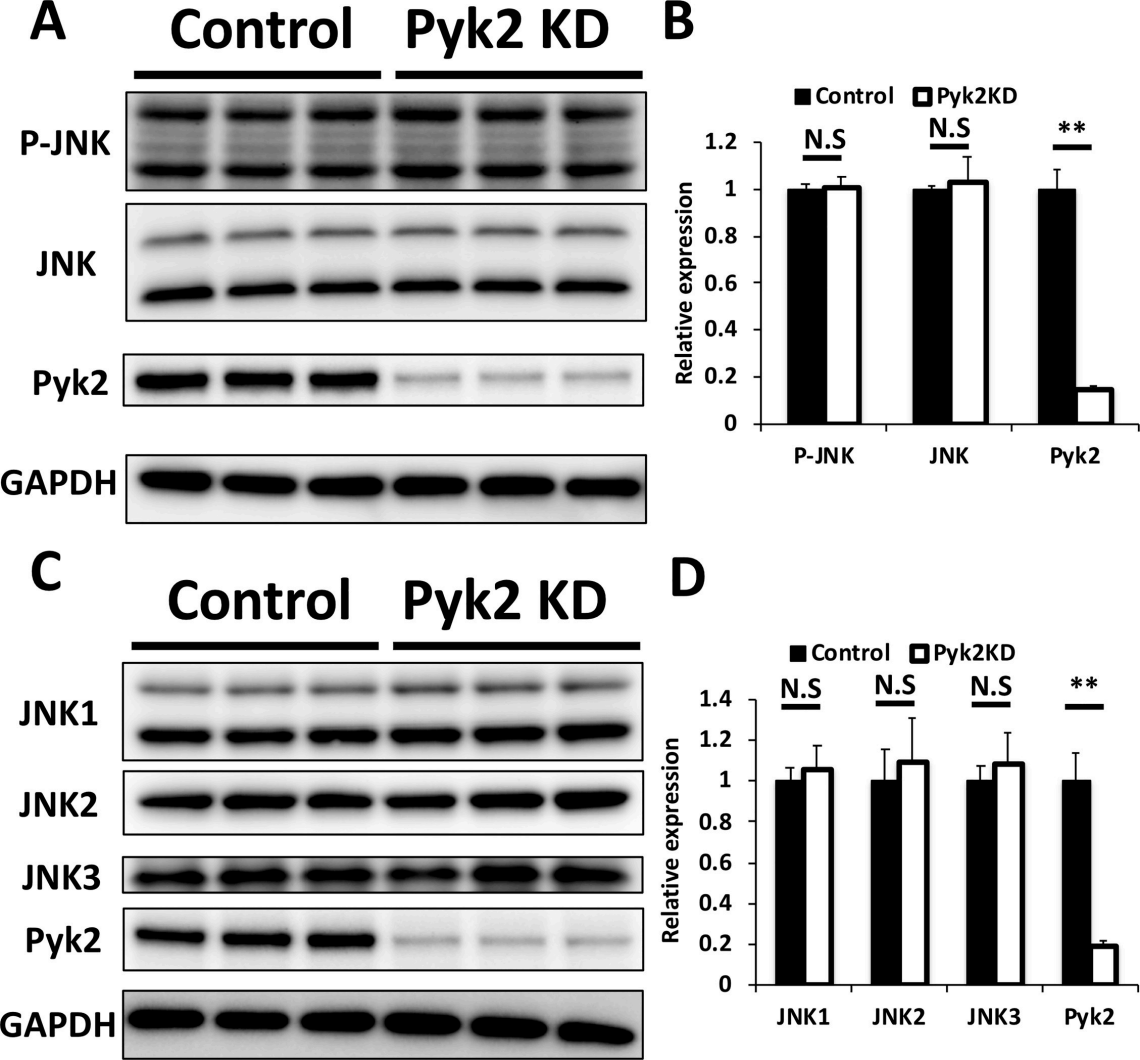
Effect of Pyk2 knockdown on JNK activation and expression. (A) EpH4-Cl3 cells were transfected with scrambled siRNA (Control) or siRNAs targeted to mRNAs encoding Pyk2 (Pyk2 KD). After 48 h, extracts from control cells or Pyk2-knockdown EpH4-Cl3 cells were subjected to immunoblotting using antibodies against phosphorylated (active) JNK (Thr-183/Tyr-185) (P-JNK), JNK, Pyk2, and GAPDH. (B) Band intensities of P-JNK, JNK, and Pyk2 in (A) were measured and normalized to GAPDH expression. The expression levels in control cells were set to 1. The means and SEMs are shown in the graph (*n* = 3; **p < 0.01; ^N.S.^*p* > 0.05). (C) EpH4-Cl3 cell extracts were prepared after the cells were treated, as described in (A), and subjected to immunoblotting using antibodies against JNK1, JNK2, JNK3, Pyk2, and GAPDH. (D) Band intensities of JNK1, JNK2, JNK3, and Pyk2 in (C) were measured and normalized to GAPDH expression. The expression levels in control cells were set to 1. The means and SEMs are shown in the graph (*n* = 3; **p < 0.01; ^N.S.^*p* > 0.05).

We previously demonstrated that JNK1 and JNK2 enhance LSR and tricellulin localization to tTJs in the mammalian epithelial cell sheet [15]. It is possible that the effect of Pyk2 knockdown might be mediated by JNK activation or overexpression. Therefore, we investigated the expression and phosphorylation levels of JNK in Pyk2-targeted siRNA- or scrambled siRNA-transfected cells by immunoblotting using antibodies against Thr-183/Tyr-185-phosphorylated JNK, JNK, and Pyk2. The expression of phosphorylated (activated) JNK and JNK was unaffected by Pyk2 knockdown (Fig 7A and 7B). Under the same conditions, JNK1, JNK2, and JNK3 expression also did not significantly change (Fig 7C and 7D). These results indicated that Pyk2-mediated localization changes of LSR and tricellulin are independent of JNK.

### Pyk2 enhanced LSR and tricellulin localization to tTJs through Tyr-237 phosphorylation of LSR

**Fig 8:**
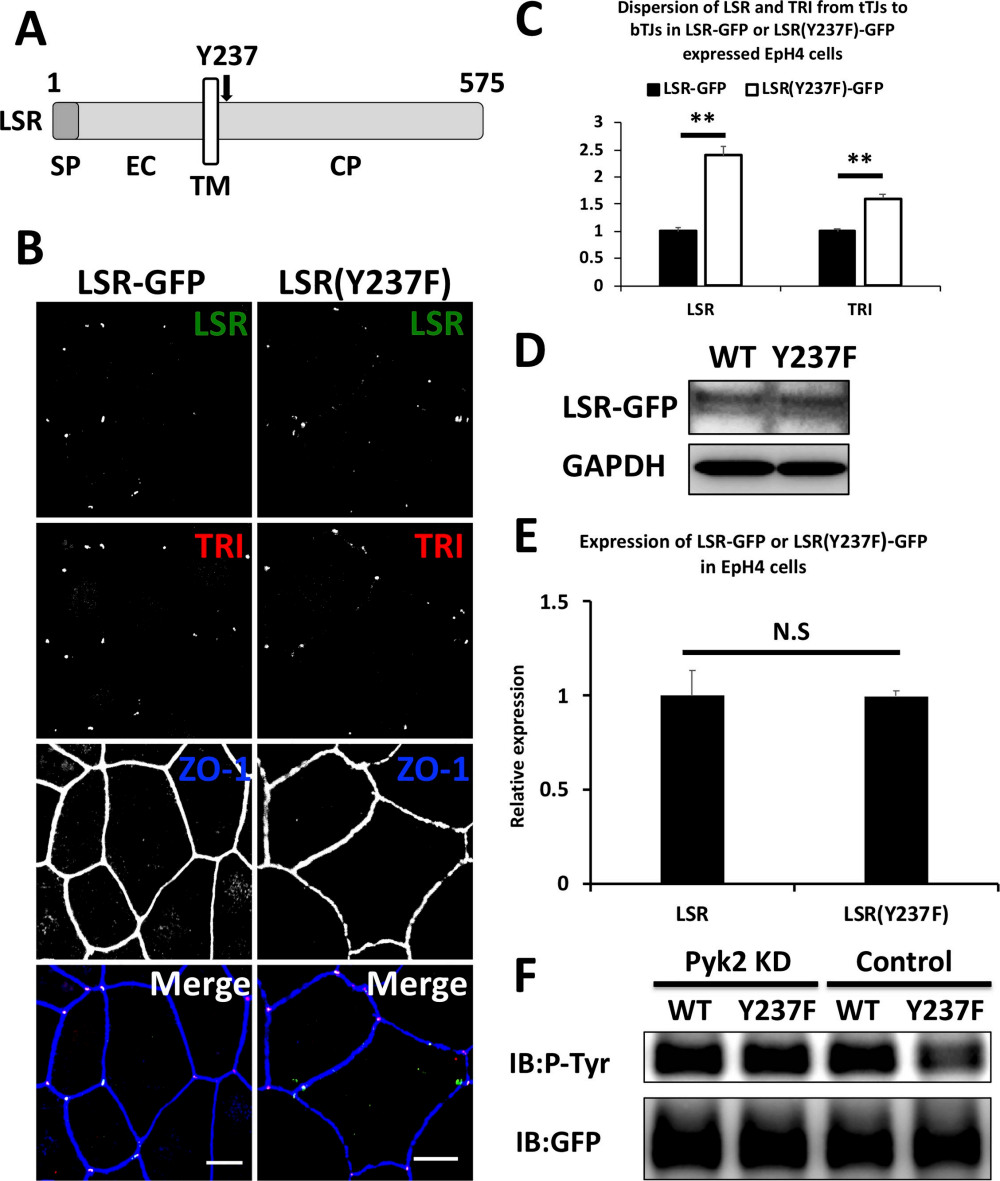
Pyk2-mediated tyrosine phosphorylation of LSR at Y237 and subcellular localization of LSR(Y237F)-GFP and tricellulin. (A) Schematic representation of the primary structure of mouse LSR. LSR has a signal peptide (SP), an extracellular domain (EC), a single transmembrane domain (TM), and a cytoplasmic domain (CP). The arrow indicates the position of Tyr-237 in the CP. (B) EpH4 cells were transfected with plasmid vectors expressing LSR-GFP or LSR(Y237F)-GFP (LSR[Y237F]) and subjected to immunofluorescence using anti-ZO-1 and anti-tricellulin (TRI) antibodies. Merge represents the merged image. Scale bar = 10 μm. (C) Dispersion of LSR and TRI in LSR-GFP or LSR(Y237F)-GFP expressing cells was calculated, and the means and SEMs are shown in the graph (*n* = 30, **p < 0.01). The dispersion values in LSR-GFP expressing cells were set to 1. (D) EpH4 cells were transfected with plasmids encoding LSR-GFP or LSR(Y237F)-GFP. The expression levels of LSR-GFP (WT) or LSR(Y237F)-GFP (Y237F) were analyzed by immunoblotting using antibodies against GFP and GAPDH. (E) The expression levels of LSR-GFP (LSR) or LSR(Y237F)-GFP (LSR[Y237F]) in (D) were quantified, and the means and SEMs are shown in the graph (*n* = 3, ^N.S.^*p* > 0.05). (F) Identification of tyrosine phosphorylation levels of immunoprecipitated LSR-GFP or LSR(Y237F)-GFP from control cells or Pyk2-knockdown EpH4 cells. Plasmid vectors expressing LSR-GFP or LSR(Y237F)-GFP were cotransfected along with scrambled siRNA (Control) or siRNAs targeted to mRNAs encoding Pyk2 (Pyk2 KD) in EpH4 cells. LSR-GFP and LSR(Y237F)-GFP were immunoprecipitated from the cells, and the expression level of total and tyrosine-phosphorylated LSR-GFP (WT) and LSR(Y237F)-GFP (Y237F) was detected by immunoblotting (IB) using anti-GFP and anti–phosphorylated tyrosine (P-Tyr) antibodies.

Masuda et al. (2014) [9] demonstrated that amino acids 1–258 of mouse LSR as well as full-length LSR were enough for localization to tTJs in EpH4 cells. Additionally, we identified that LSR and tricellulin localization in the cells is regulated by the phosphorylation state of LSR [15]. These results suggested that phosphorylation of amino acids 1–258 of LSR might be important for LSR and tricellulin localization to tTJs. Additionally, we predicted the phosphorylation site of LSR by Pyk2 using GPS3.0, a method of sequence-based prediction of kinase-specific phosphorylation sites (http://gps.biocuckoo.org/online.php) [24] and identified possible phosphorylation sites as tyrosine 237 and tyrosine 447. From these results, we speculated that Pyk2-dependent phosphorylation of LSR at Tyr-237 might be responsible for LSR and tricellulin localization to tTJs (Fig 8A).

**Fig 9:**
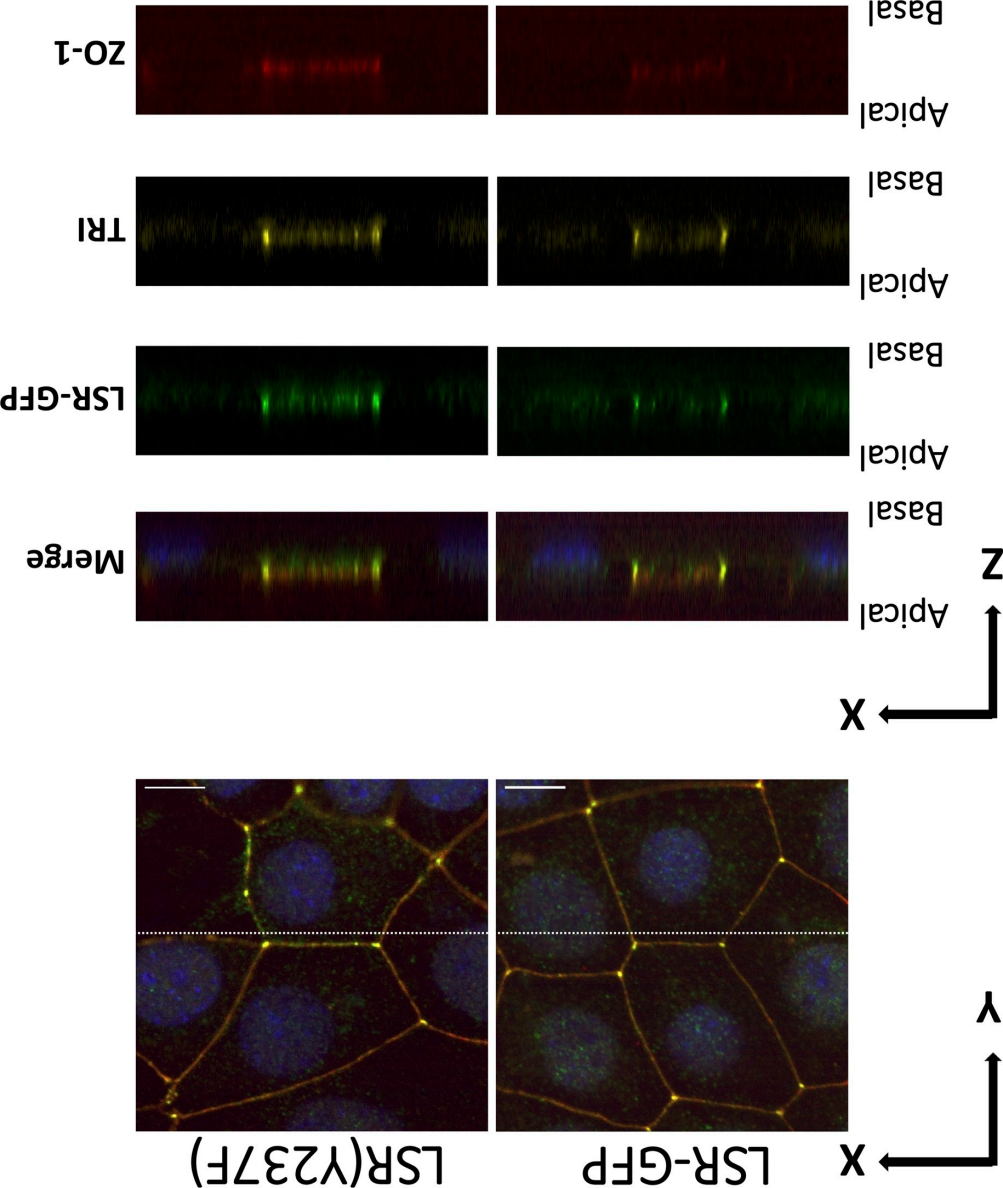
Three-dimensional subcellular localization of LSR and tricellulin in LSR-GFP or LSR(Y237F)-GFP expressed cells. EpH4 cells were treated under the same conditions as in Fig 8B. The cells were then immunostained with anti-tricellulin (TRI) and ZO-1 antibodies and counterstained with DAPI, and three-dimensional images of the cells were prepared as described in the Materials and Methods. We identified the three-dimensional subcellular localization of LSR-GFP, LSR(Y237F)-GFP and TRI in LSR-GFP or LSR(Y237F)-GFP expressed cells using the images. Imaging along the z-axis at the indicated position (white dotted line, upper panel) reveals colocalization of LSR(Y237F)-GFP and TRI with ZO-1 at bTJs in LSR(Y237F)-GFP expressed cells (lower panel). Bar, 10 μm.

To test whether Tyr-237 dephosphorylation is critical for LSR and tricellulin localization, we examined the localization of GFP-conjugated LSR(Y237F), a mutant with Tyr-237 substituted with phenylalanine, using confocal microscopy. EpH4 cells were transfected with plasmids encoding LSR‐GFP or LSR(Y237F)‐GFP. After 3 days of the transfection, the cells were subjected to immunofluorescence using anti-tricellulin and anti-ZO‐1 antibodies. Immunofluorescence of ZO-1 was performed for visualization of tTJs and bTJs. In LSR‐GFP-expressing cells, LSR‐GFP and tricellulin specifically localized to tTJs (Fig 8B, LSR-GFP). In contrast, these tTJ proteins diffused from tTJs into bTJs and cytoplasmic regions in LSR(Y237F)‐GFP‐expressing cells (Fig 8B, LSR[Y237F]). It was interesting to note that tricellulin was also diffused to the bTJs and cytoplasmic regions, even in the presence of the endogenous LSR (Fig 8B, LSR[Y237F]). Quantification of these results, as described in the Materials and Methods section, confirmed significant dispersal of LSR and tricellulin to bTJs in cells expressing LSR(Y237F)-GFP (Fig 8C). We confirmed by immunoblotting analysis that the expression of LSR-GFP and LSR(Y237F)-GFP is similar (Fig 8D and 8E). This indicates that the dispersal of LSR(Y237F)-GFP to the bTJs sites was not due to overexpression of LSR(Y237F)-GFP. Subsequently, three-dimensional imaging analysis showed that LSR and tricellulin were mainly diffused as dot structures from tTJs into bTJs in LSR(Y237F)-GFP expressed cells as well as in PF-43 treated or Pyk2 knockdown cells (Fig 9). These results suggest that phosphorylation of LSR at tyrosine 237 is essential for the localization not only of LSR but also of tricellulin at the tTJs.

Next, to investigate the tyrosine phosphorylation level of LSR‐GFP or LSR(Y237F)‐GFP in cells, we expressed LSR‐GFP or LSR(Y237F)‐GFP in Pyk2‐knockdown or control EpH4 cells. We performed immunoprecipitation using anti-GFP antibody and, subsequently, immunoblotting using anti-phospho-tyrosine and anti-GFP antibodies to examine tyrosine phosphorylation levels of LSR‐GFP and LSR(Y237F)-GFP (Detailed experimental conditions are described in Materials and Methods). The tyrosine phosphorylation level of LSR(Y237F)‐GFP was found to be decreased compared to LSR‐GFP in control cells (Fig 8F, WT), suggesting that Tyr-237 is phosphorylated under normal conditions. In contrast, the tyrosine phosphorylation level was comparable between LSR‐GFP and LSR(Y237F)‐GFP in Pyk2-knockdown cells (Fig 8F, Y237). Unexpectedly, the phosphorylation levels of LSR‐GFP and LSR(Y237F)‐GFP in Pyk2‐knockdown cells were not decreased compared to LSR‐GFP in control cells (Fig 8F) but rather seemed similar. We assumed that Pyk2 is responsible for not only phosphorylation at Tyr-237 but also dephosphorylation of other tyrosine sites. These results suggested that LSR and tricellulin localization to tTJs is regulated by tyrosine phosphorylation of LSR at Tyr-237, which is mediated by Pyk2. The direct interaction between LSR-GFP and Pyk2 could not be detected by co-immunoprecipitation assay (S4 Fig, see Discussion).

### Pyk2 knockdown or LSR dephosphorylation at Tyr-237 but not PF-43 treatment enhanced epithelial barrier function

**Fig 10:**
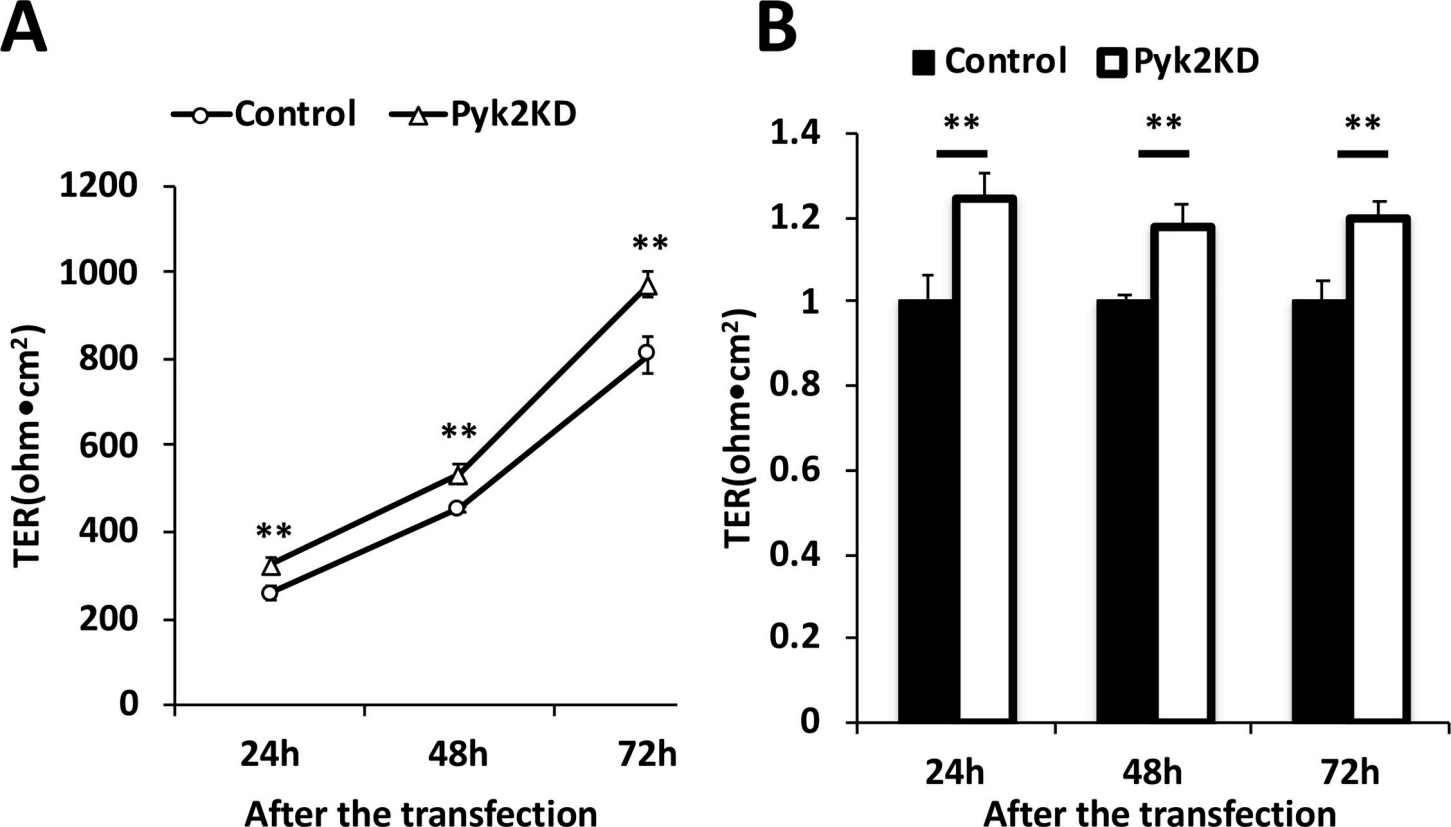
Effects of Pyk2 knockdown on epithelial barrier function. The epithelial barrier function of EpH4-Cl3 cells was evaluated by measuring the TER. (A) EpH4-Cl3 cells were transfected with scrambled siRNA (Control) or siRNAs targeted to mRNAs encoding Pyk2 (Pyk2 KD). After 24, 48, and 72 h, TER of control cells or Pyk2-knockdown EpH4-Cl3 cells was measured (*n* = 6 for each cell line; **p < 0.01). (B) The TER of control and Pyk2-knockdown cells in (A) was quantified, and the means and SEMs are shown in the graph (*n* = 6; **p < 0.01).

**Fig 11:**
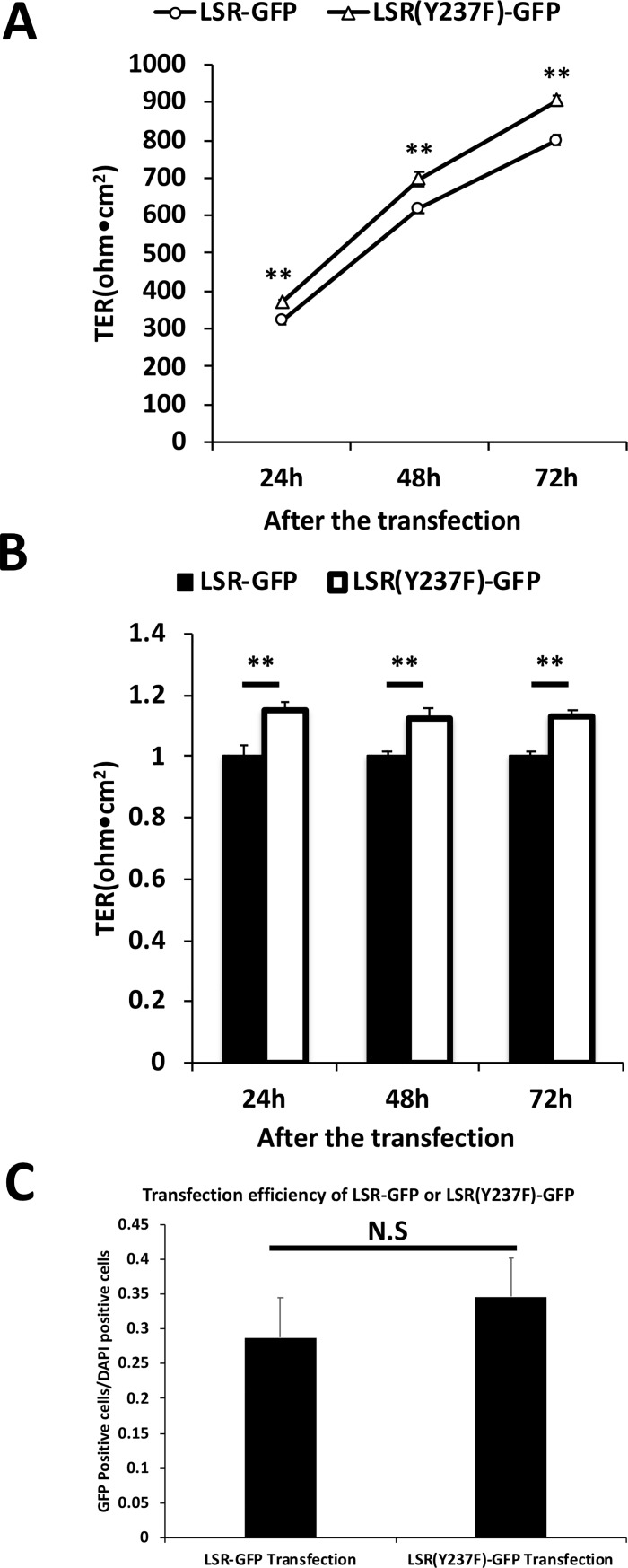
Effects of LSR(Y237F)-GFP on epithelial barrier function. The epithelial barrier function of EpH4 cells was evaluated by measuring the TER. EpH4 cells were transfected with plasmid vectors expressing LSR-GFP or LSR(Y237F)-GFP. (A) After 24, 48, and 72 h, the TER of LSR-GFP- or LSR(Y237F)-GFP-expressing EpH4 cells was measured (*n* = 6 for each cell line; **p < 0.01). (B) The TER of LSR-GFP- and LSR(Y237F)-GFP-expressing EpH4 cells in (A) was quantified, and the means and SEMs are shown in the graph (*n* = 6; **p < 0.01). (C) At 24 h after the transfection, transfection efficiency of LSR-GFP- or LSR(Y237F)-GFP in EpH4 cells was measured by counting the percentage of GFP-positive cells for DAPI positive cells by microscopic observation (*n* = 5–6 for each cell line; ^N.S.^*p* > 0.05).

Overexpression of tricellulin in tTJs and bTJs has different effects on the epithelial barrier function of the mammalian epithelial cell sheet [25], suggesting that mislocalization of tTJ proteins from tTJs to other regions influences epithelial barrier function. Therefore, we examined the effects of LSR or tricellulin mislocalization by PF-43 treatment, Pyk2 knockdown or LSR(Y237F) expression on the epithelial barrier function. The barrier function was assessed by TER measurements. PF-43 caused the significant loss of TER as the treatment perturbed the structural integrity of both bTJs and tTJs (S5 Fig). At 24, 48, or 72 h after siRNA transfection, TER was significantly increased in Pyk2-knockdown EpH4-Cl3 cells compared to scrambled siRNA–transfected cells (Fig 10A and 10B). At the same time points, LSR(Y237F)-GFP overexpression also significantly increased TER compared to LSR-GFP overexpression (Fig 11A and 11B), although the difference in TER between LSR-GFP and LSR(Y237F)-GFP expressing cells was smaller than between scramble- and Pyk2-siRNA transfected cells. This was likely due to the reduced transfection efficiency of the plasmid (28.7±5.7% or 34.6±5.5% in LSR-GFP- or LSR(Y237F)-GFP-transfected cells) compared to the almost complete loss of Pyk2 protein by RNAi (Fig 11C). These results suggested that specific localization of LSR and tricellulin to tTJs depends on phosphorylation of LSR at Tyr-237 and inhibits epithelial barrier function.

## Discussion

### Pyk2-dependent LSR phosphorylation at Tyr-237 enhances LSR and tricellulin localization to tTJs

LSR is a hyperphosphorylated protein that has at least 30 phosphorylation sites in human and mouse cells (https://www.phosphosite.org/) [26]. Phosphorylation of LSR modulates protein–protein interactions and subcellular localization of LSR. For example, we identified previously that mouse LSR phosphorylation at Ser-288 enhances localization of LSR and tricellulin (a downstream tTJ protein) to tTJs in EpH4 cells [15]. Dubois et al. (2009) [27] demonstrated that human LSR phosphorylation at Ser-493 contributes to protein–protein interactions between the LSR and 14-3-3 proteins in the human embryonic kidney cell line HEK293. However, the role of many phosphorylation sites in LSR is still not well understood. In this study, we demonstrated that phosphorylation of mouse LSR at Tyr 237 is required for proper localization of LSR and tricellulin at tTJs and the phosphorylation is dependent on activation of non-receptor tyrosine kinase Pyk2 (Figs 8B, 8C, 8F and 9). More interestingly, the tyrosine phosphorylation level of LSR(Y237F)-GFP was decreased compared with LSR-GFP in the control cells (Fig 8F), while the phosphorylation levels of LSR-GFP and LSR(Y237F)-GFP in Pyk2-knockdown cells were not decreased compared to LSR-GFP in control cells (Fig 8F). These results suggested that Pyk2 knockdown enhances tyrosine phosphorylation of LSR except for Tyr-237. This suggestion is supported by previous studies demonstrating that Pyk2 activates other tyrosine kinases, such as Src and epidermal growth factor receptor (EGFR), and that it interacts with many protein tyrosine phosphatases, suggesting that Pyk2 knockdown induces various changes in the tyrosine phosphorylation pattern of LSR [28–35].

On the other hand, LSR(Y237F)-GFP diffused from tTJs into bTJs and cytoplasmic regions (Figs 8B, 8C and 9), indicated that phosphorylation at Tyr-237 but not at other tyrosine sites is essential for LSR localization to tTJs. Under the same conditions, tricellulin also diffused from tTJs into bTJs and cytoplasmic regions (Figs 8B, 8C and 9), suggesting that LSR phosphorylation at Tyr-237 does not inhibit protein–protein interactions between LSR and tricellulin. We observed the mislocalization of these tTJ proteins in Pyk2-knockdown or Pyk2-inactivated EpH4-Cl3 cells (Figs 1, 2, 4 and 6), too, indicating that tyrosine phosphorylation of LSR, except for Tyr-237, in Pyk2-dysfunctional cells does not inhibit mislocalization. We previously identified that LSR dephosphorylation at Ser-288 inhibits LSR and tricellulin localization to tTJs [15]. Therefore, although we cannot rule out the possibility that Pyk2-dependent phosphorylation of LSR at Tyr-237 accumulates LSR and tricellulin at tTJs through LSR phosphorylation at Ser-288, we consider this hypothesis unlikely because Pyk2 knockdown had no significant effect on JNK activation and expression (Fig 7), which is an essential factor for LSR phosphorylation at Ser-288 in EpH4 cells [15].

However, the question remains as to which kinase directly phosphorylates LSR at Tyr-237. To investigate the possibility of direct phosphorylation of LSR at Tyr-237 by Pyk2, we validated the protein–protein interactions between LSR and Pyk2 using co-immunoprecipitation assay; however, unfortunately, we could not detect this interaction in EpH4 cells (S4 Fig). Maybe, to identify kinase–substrate interactions, detection using a highly sensitive assay, such as the AlphaScreen, is required [36,37]. Additionally, Pyk2 might activate LSR phosphorylation at Tyr-237 by other tyrosine kinases. For example, prediction by GPS3.0 showed that LSR at Tyr-237 is more likely to be directly phosphorylated by Axl, Fer, and Txk and Pyk2 activates other tyrosine kinases, such as Src and EGFR [28,33–35]. Therefore, identifying tyrosine kinases directly phosphorylating LSR at Tyr-237 is a key challenge for the future.

### Dispersion of LSR and tricellulin from tTJs to bTJs enhances epithelial barrier function

Previous studies suggested that dispersion of tTJs proteins from tTJs to other regions induces the disruption of tTJs and resulting in abnormal epithelial barrier function [9,11,25]. Therefore, we analyzed effect of Pyk2-knockdown or LSR(Y237F) expressing, which induce mislocalization of tTJ proteins LSR and tricellulin from tTJs to bTJs and cytoplasmic regions (Figs 4, 6, 8B, 8C and 9), on epithelial barrier function, and indicated that epithelial barrier function is impaired under these conditions (Figs 10 and 11). These results are different from a previous study that reported that in tricellulin^R497X/R497X^ mice, paracellular permeability properties of the stria vascularis are unaffected when tricellulin is dispersed from tTJs to other regions [38]. Another study reported that in Madin–Darby canine kidney II (MDCK II) cells, an increase in tricellulin in bTJs through transfection of an expression vector enhances epithelial barrier function [25]. These results suggested that the effect of LSR and tricellulin relocalization from tTJs to bTJs on epithelial barrier function is controversial. Our results support the second model, in which dispersion of tTJ proteins from tTJs to bTJs enhances epithelial barrier function.

The effects of LSR dispersion in bTJs on the epithelial barrier function of the mammalian epithelial cell sheet vary by cell type and tissue. We hypothesized that the reason is the differences in LSR interaction partners in bTJs. This hypothesis is supported by studies that bTJs in each tissue comprise tissue-specific combinations of proteins [39,40] and that LSR interacts with specific bTJ proteins (e.g., occludin) but not others (e.g., marveld3) [10]. Therefore, combinations of bTJ proteins influenced by LSR dispersion in bTJs vary by cell type and tissue, and the resulting epithelial barrier function of the mammalian epithelial cell sheet causes tissue- or cell-specific changes.

LSR interacts with not only bTJ proteins but also cytoplasmic protein (e.g., MAPKAPK5) (https://thebiogrid.org/) [41]. Therefore, LSR phosphorylation might affect its interaction with bTJ and other cytoplasmic proteins. This hypothesis was supported by the above-mentioned study showing that human LSR phosphorylation at Ser-493 enhances protein–protein interactions between LSR and 14-3-3 proteins [27]. According to Dubois et al. (2009) [27], LSR dispersion in bTJs in Tyr-237-dephosphorylated or Pyk2-dysfunctional EpH4 cells might be influenced by changes in the strength of phosphorylation-dependent protein–protein interactions. Elucidating phosphorylation-dependent interacting partners would contribute to our understanding of the molecular mechanisms of how tTJ proteins are recruited to tTJs and how this results in changing epithelial barrier function.

LSR also functions as an activator for the intracellular uptake of apolipoprotein E (ApoE) and apolipoprotein B (ApoB) [42,43]. These proteins may affect epithelial barrier integrity by regulating the expression of claudin proteins. For example, ApoE can positively regulate epithelial barrier function and increase claudin-5 expression [44–45], which reportedly enhances barrier function [46]. ApoB might also be involved in barrier integrity via the decreased expression of claudin-2, which reportedly suppresses barrier function [47,48], via regulating Akt [48–50]. Therefore, it would be interesting to examine the uptake of ApoE or ApoB when LSR is dispersed to bTJs, indicating that not only the tight interactions of tTJ proteins but also the regulation of the intracellular uptake of these molecules could contribute to epithelial barrier function.

## Conclusions

Pyk2 enhances LSR and tricellulin localization to tTJs. This recruitment is performed by Pyk2-dependent phosphorylation of LSR at Tyr-237 and inhibits epithelial barrier function. Our findings indicated a novel mechanism by which Pyk2 regulates tTJ assembly and epithelial barrier function in the mammalian epithelial cell sheet.

## Supporting information

S1 Fig Double immunostaining of LSR and tricellulin in EpH4-Cl3 cells. EpH4-Cl3 cells were double immunostained with anti-LSR and anti-tricellulin (TRI) antibodies, and observed using confocal microscopy. Merge represents the merged image. Scale bar = 10 μm.
(TIF)

S2 Fig Effect of PF-43 treatment on EpH4-Cl3 cells. EpH4-Cl3 cells were incubated with PF-43 (10, 20, 40 or 80 μM) or DMSO (Control) for 2 h (A) or with 20 μM PF-43 or DMSO for 4 h (B). The cells were then immunostained with anti-LSR antibody. Merge represents the merged image. Bar = 10 μm.
(TIF)

S3 Fig FAK and Pyk2 activation and subcellular localization of tTJ proteins in GSK2256098-treated EpH4-Cl3 cells. EpH4-Cl3 cells were incubated with DMSO (Control) or 1–80 μM GSK2256098 (GSK) for 120 min. The extracts were subjected to immunoblotting using antibodies against phosphorylated FAK (Tyr397) (P-FAK) (A), phosphorylated Pyk2 (Tyr402) (P-Pyk2) (B) and GAPDH. (C) Band intensities of P-FAK in (A) and P-Pyk2 in (B) were measured and normalized to GAPDH expression. The expression levels in control cells were set to 1. EpH4-Cl3 cells were incubated with DMSO (Control) or 1 μM GSK for 120 min. The cells were then immunostained with anti-LSR (C, LSR) and anti-tricellulin (D, TRI) antibodies, and observed using confocal microscopy. The red rectangular regions represent higher magnifications (LSR-High and TRI-High). Merge represents the merged image. Scale bar = 10 μm.
(TIF)

S4 Fig Interaction of LSR-GFP and Pyk2 in EpH4 cells. Detection of the interaction between LSR-GFP and Pyk2 in EpH4 cells was carried out as described previously [15]. EpH4 cells were transfected with plasmids encoding LSR-GFP. After 72 h, the cell lysates were prepared and immunoprecipitated (IP) with anti-GFP or normal rabbit IgG (IgG) antibody, followed by immunoblotting analysis using anti-GFP or Pyk2 antibody.
(TIF)

S5 Fig Effects of PF-43 treatment on epithelial barrier function. The epithelial barrier function of EpH4-Cl3 cells was evaluated by measuring the TER. (A) EpH4-Cl3 cells were cultured for 24 h and after incubated with DMSO (Control) or 20 μM PF-43. At 24, 48, and 72 h after the incubation, TER of control or PF-43-treated cells was measured (*n* = 6 for each cell line). (B) The TER of control and PF-43-treated cells in (A) was quantified, and the means and SEMs are shown in the graph (*n* = 6; **p < 0.01; ^N.S.^*p* > 0.05).
(TIF)
