# Supplementary figures and images for: Pyk2-dependent phosphorylation of LSR enhances localization of LSR and tricellulin at tricellular tight junctions
Source: PLoS One. 2019 Oct 1;14(10):e0223300. doi: 10.1371/journal.pone.0223300 (PMC6773211; doi:10.1371/journal.pone.0223300)

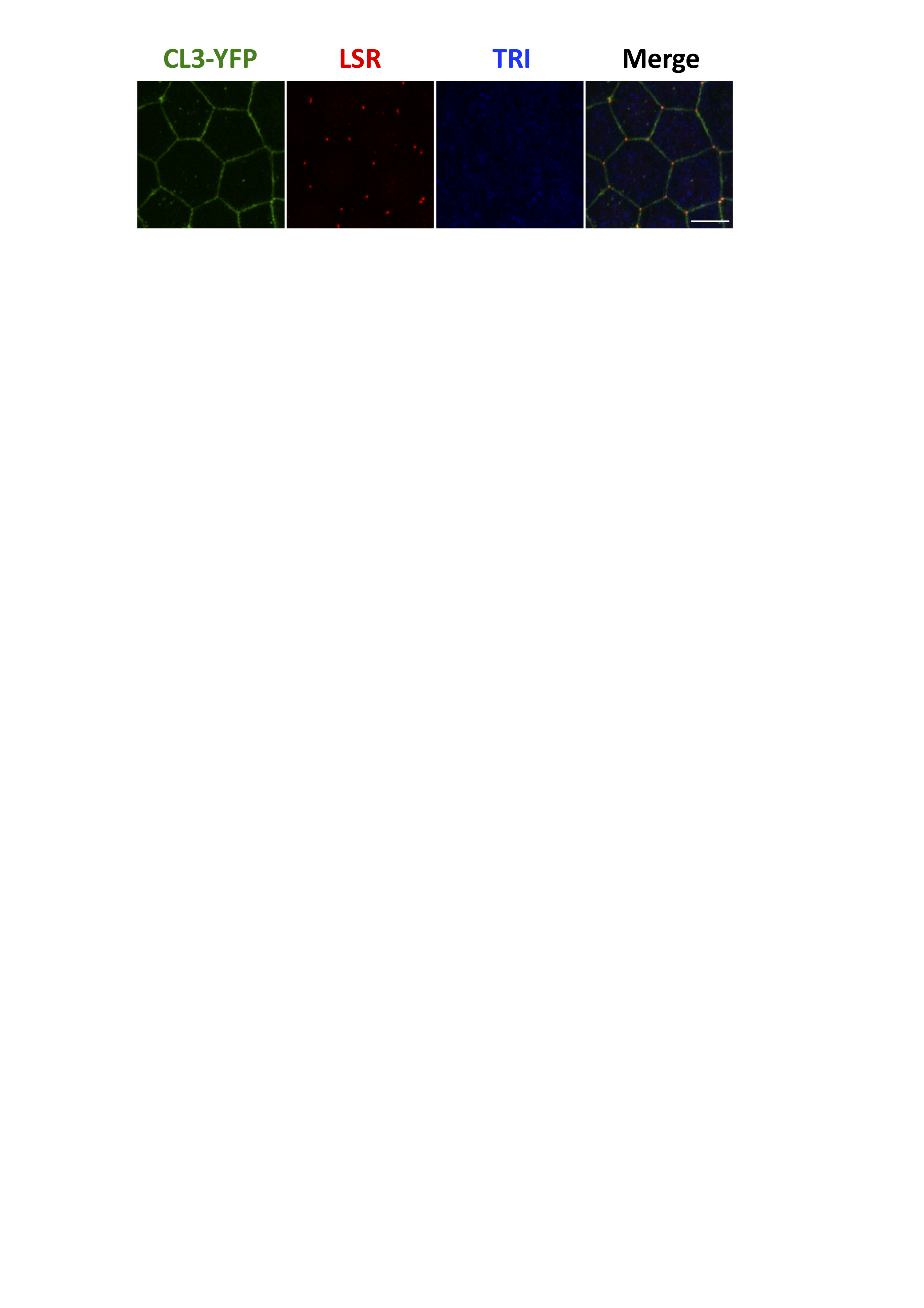

Supplement: S1 Fig — EpH4-Cl3 cells were double immunostained with anti-LSR and anti-tricellulin (TRI) antibodies, and observed using confocal microscopy. Merge represents the merged image. Scale bar = 10 μm. (TIF) [file pone.0223300.s001.tif]

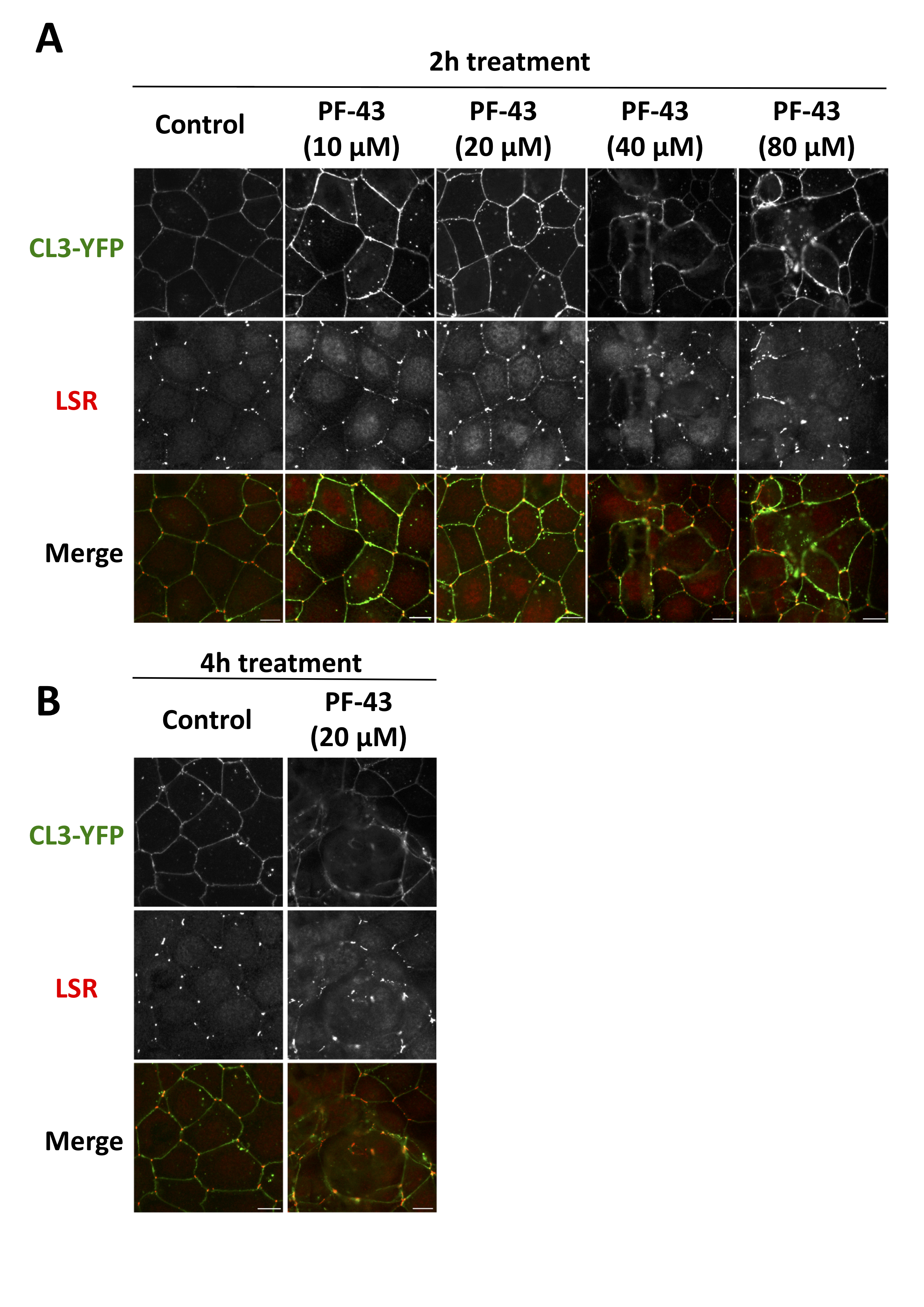

Supplement: S2 Fig — EpH4-Cl3 cells were incubated with PF-43 (10, 20, 40 or 80 μM) or DMSO (Control) for 2 h (A) or with 20 μM PF-43 or DMSO for 4 h (B). The cells were then immunostained with anti-LSR antibody. Merge represents the merged image. Bar = 10 μm. (TIF) [file pone.0223300.s002.tif]

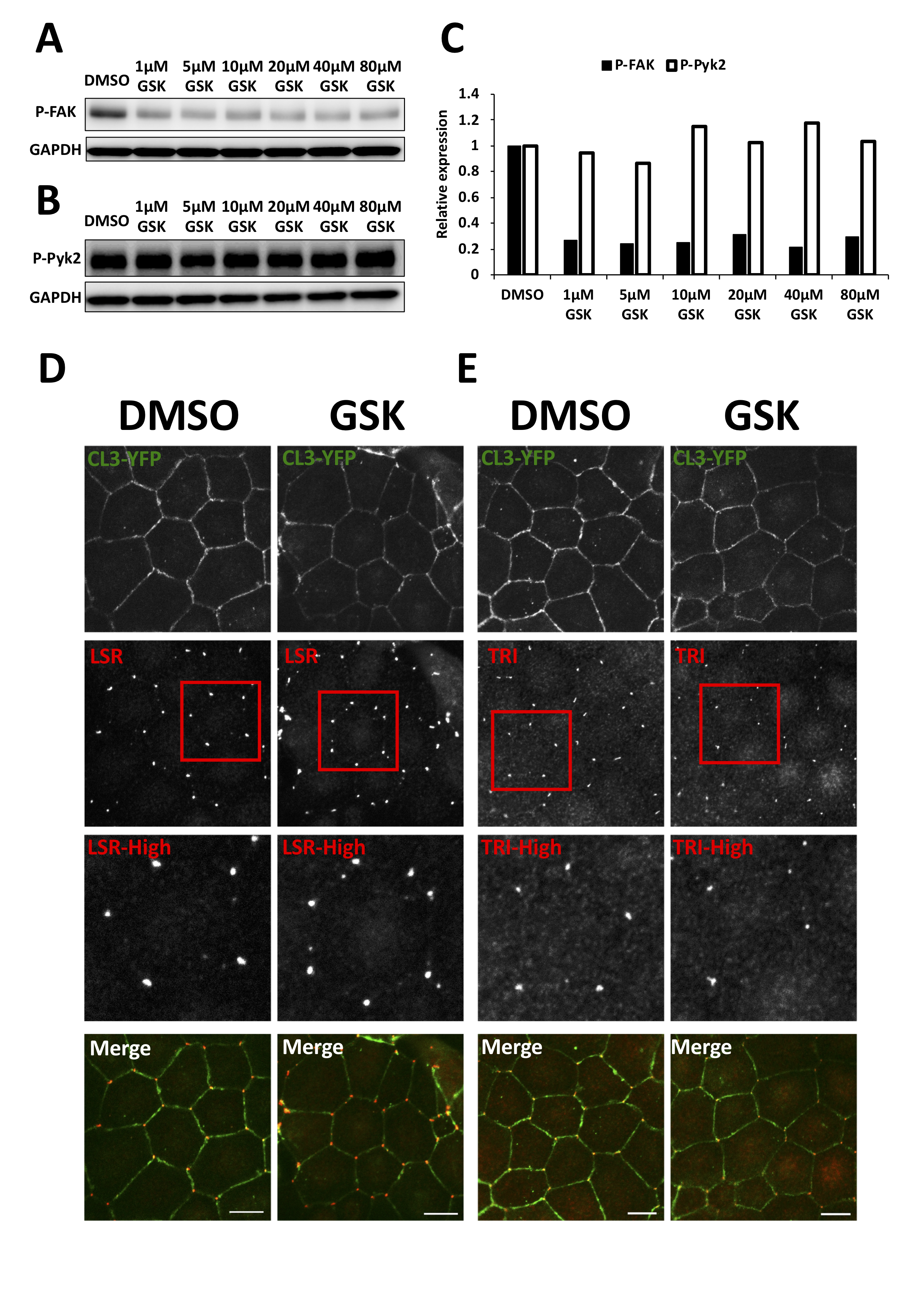

Supplement: S3 Fig — EpH4-Cl3 cells were incubated with DMSO (Control) or 1–80 μM GSK2256098 (GSK) for 120 min. The extracts were subjected to immunoblotting using antibodies against phosphorylated FAK (Tyr397) (P-FAK) (A), phosphorylated Pyk2 (Tyr402) (P-Pyk2) (B) and GAPDH. (C) Band intensities of P-FAK in (A) and P-Pyk2 in (B) were measured and normalized to GAPDH expression. The expression levels in control cells were set to 1. EpH4-Cl3 cells were incubated with DMSO (Control) or 1 μM GSK for 120 min. The cells were then immunostained with anti-LSR (C, LSR) and anti-tricellulin (D, TRI) antibodies, and observed using confocal microscopy. The red rectangular regions represent higher magnifications (LSR-High and TRI-High). Merge represents the merged image. Scale bar = 10 μm. (TIF) [file pone.0223300.s003.tif]

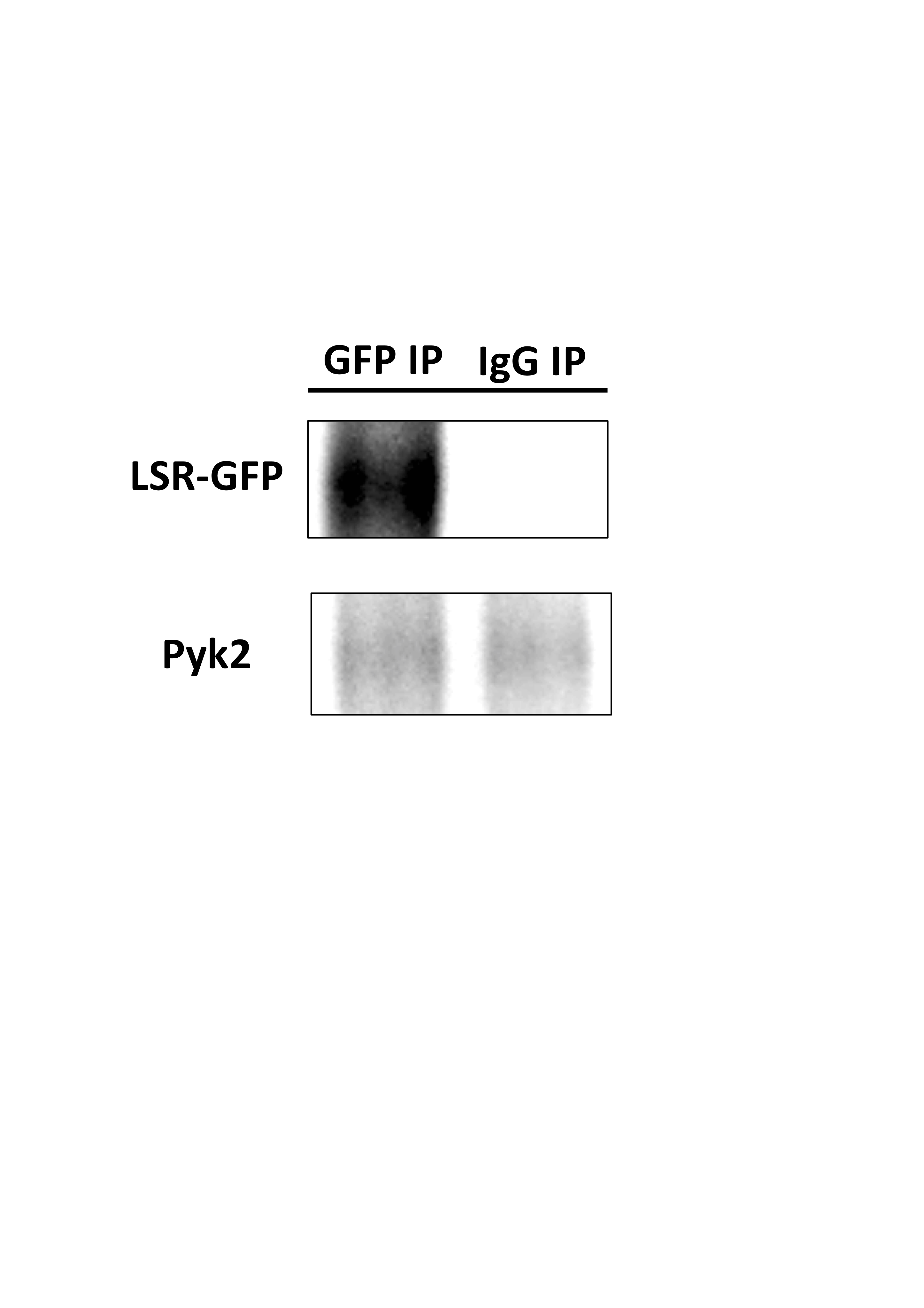

Supplement: S4 Fig — Detection of the interaction between LSR-GFP and Pyk2 in EpH4 cells was carried out as described previously [15]. EpH4 cells were transfected with plasmids encoding LSR-GFP. After 72 h, the cell lysates were prepared and immunoprecipitated (IP) with anti-GFP or normal rabbit IgG (IgG) antibody, followed by immunoblotting analysis using anti-GFP or Pyk2 antibody. (TIF) [file pone.0223300.s004.tif]

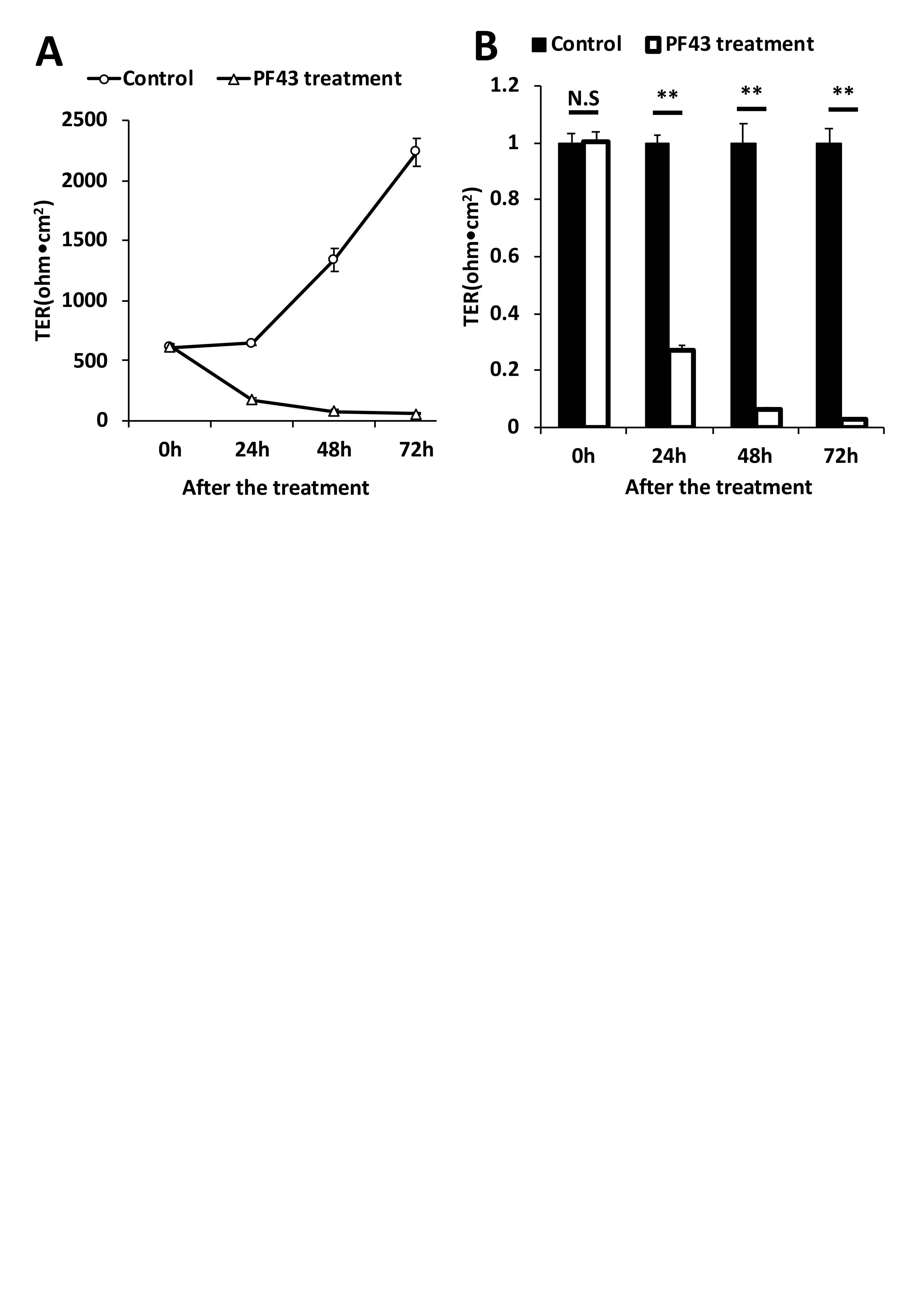

Supplement: S5 Fig — The epithelial barrier function of EpH4-Cl3 cells was evaluated by measuring the TER. (A) EpH4-Cl3 cells were cultured for 24 h and after incubated with DMSO (Control) or 20 μM PF-43. At 24, 48, and 72 h after the incubation, TER of control or PF-43-treated cells was measured (n = 6 for each cell line). (B) The TER of control and PF-43-treated cells in (A) was quantified, and the means and SEMs are shown in the graph (n = 6; **p < 0.01; N.S.p > 0.05). (TIF) [file pone.0223300.s005.tif]
